# Italian guidelines on the assessment and management of pediatric head injury in the emergency department

**DOI:** 10.1186/s13052-017-0442-0

**Published:** 2018-01-15

**Authors:** Liviana Da Dalt, Niccolo’ Parri, Angela Amigoni, Agostino Nocerino, Francesca Selmin, Renzo Manara, Paola Perretta, Maria Paola Vardeu, Silvia Bressan, on behalf of the Italian Society of Pediatric Emergency Medicine (SIMEUP), the Italian Society of Pediatrics (SIP)

**Affiliations:** 1 Pediatric Emergency Department-Intensive Care Unit, Department of Woman’s and Child’s Health, University of Padova, Via Giustiniani 2, 35128 Padova, Italy; 2 Department of Pediatric Emergency Medicine and Trauma Center, Meyer University Children’s Hospital, Florence, Italy; 3 Department of Pediatrics, S. Maria della Misericordia University Hospital, University of Udine, Udine, Italy; 4 Department of Radiology, Neuroradiology Unit, University of Salerno, Salerno, Italy; 5 Neurosurgery Unit, Regina Margherita Pediatric Hospital, Torino, Italy; 6 Pediatric Emergency Department, Regina Margherita Pediatric Hospital, Torino, Italy

**Keywords:** Children, Traumatic brain injury, Emergency department, Evidence-based, Guideline

## Abstract

**Objective:**

We aim to formulate evidence-based recommendations to assist physicians decision-making in the assessment and management of children younger than 16 years presenting to the emergency department (ED) following a blunt head trauma with no suspicion of non-accidental injury.

**Methods:**

These guidelines were commissioned by the Italian Society of Pediatric Emergency Medicine and include a systematic review and analysis of the literature published since 2005. Physicians with expertise and experience in the fields of pediatrics, pediatric emergency medicine, pediatric intensive care, neurosurgery and neuroradiology, as well as an experienced pediatric nurse and a parent representative were the components of the guidelines working group.

Areas of direct interest included 1) initial assessment and stabilization in the ED, 2) diagnosis of clinically important traumatic brain injury in the ED, 3) management and disposition in the ED. The guidelines do not provide specific guidance on the identification and management of possible associated cervical spine injuries. Other exclusions are noted in the full text.

**Conclusions:**

Recommendations to guide physicians practice when assessing children presenting to the ED following blunt head trauma are reported in both summary and extensive format in the guideline document.

## Summary of recommendations

### Initial assessment and stabilization

Clinicians must follow the ABCDE approach according to the ATLS/PALS/EPALS principles for the initial assessment and management of all children with severe head trauma (Evidence Quality: X; Recommendation Strength: Strong Recommendation)
In children presenting to the ED with severe blunt head trauma and with signs of raised intracranial pressure (ICP) administration of hyperosmolar therapy with hypertonic saline should be considered (Evidence Quality: B; Recommendation Strength: Moderate Recommendation)
a) Clinicians should avoid hyperventilation in children presenting to the ED with signs of ICP following a severe head trauma (Evidence Quality: C; Recommendation Strength: Moderate Recommendation);
  b) In children presenting to the ED with signs of impending cerebral herniation following severe head trauma, clinicians may consider hyperventilation as a temporary measure to rapidly reduce ICP in order to increase the patient chances of undergoing a life-saving intervention (Evidence Quality: D; Recommendation Strength: Weak Recommendation)
In children presenting to the ED with severe blunt head trauma, steroids should not be administered (Evidence Quality: B; Recommendation Strength: Strong Recommendation)
In children presenting with severe blunt head trauma, hypothermia should not be initiated in the ED (Evidence Quality: A; Recommendation Strength: Strong Recommendation)

### Diagnosis of clinically important traumatic brain injury

#### CT scan decision-making

6. a) Physicians should perform a head CT in all head injured children presenting to the ED with a GCS < 14 (Evidence Quality: A; Recommendation Strength: Strong Recommendation)
  b) Physicians should use the age-appropriate PECARN algorithms to assist their decision-making about head CT scan in children with a GCS ≥ 14 (Evidence Quality: A; Recommendation Strength: Strong Recommendation).
  c) Physicians should favor initial observation over CT scan for children at intermediate-risk for clinically important traumatic brain injury (ciTBI) according to the age-appropriate PECARN algorithms, especially in the presence of isolated findings (Evidence Quality: A; Recommendation Strength: Strong Recommendation)
7. In children with ventricular shunt who sustain a minor head trauma and have no PECARN predictors of traumatic brain injury and no other risk factors from history, clinicians should favor initial observation over routine immediate CT scan (Evidence Quality: B; Recommendation Strength: Moderate Recommendation)

#### Repeat CT scan

8. Clinicians should avoid routine repeat CT scan in children with GCS 14–15 and a non-clinically significant intracranial injury on initial CT. Decision on repeating CT should be based on a careful monitoring of the neurological status and consultation with the neurosurgeon (Evidence Quality: C; Recommendation Strength: Weak Recommendation)

#### Other imaging

9. In children presenting to the ED with minor head trauma clinicians should not use skull radiographs as a screening tool for clinically important traumatic brain injuries. (Evidence Quality: B; Recommendation Strength: Strong Recommendation)
10. a) Clinicians should not routinely use trans-fontanelle ultrasound for diagnosing intracranial injuries in infants presenting to the emergency department following a trauma to the head (Evidence Quality: D; Recommendation Strength: Weak Recommendation)
  b) Clinicians may choose to use point-of-care ultrasound for the identification of skull fractures and the definition of their characteristics (e.g. depression, diastasis) in children with minor head trauma (Evidence Quality: B; Recommendation Strength: Moderate Recommendation)
11. Clinicians should not routinely use near-infrared spectroscopy (NIRS) technology devices to screen for intracranial hematomas in the assessment of children presenting to the emergency department following a trauma to the head. (Evidence Quality: C; Recommendation Strength: Weak Recommendation)

### Management and disposition

#### Observation

12. a) ED physicians should favor initial observation over CT scan for children at intermediate-risk of clinically important traumatic brain injury (ciTBI) according to the age-appropriate PECARN algorithms, especially in the presence of isolated findings. (Evidence Quality: B; Recommendation Strength: Strong Recommendation)
  b) ED physicians who elect to observe previously-healthy children >3 months of age at PECARN intermediate risk of ciTBI following a minor head trauma, should observe these patients for a minimum of 4–6 h from the time of injury. (Evidence Quality: C; Recommendation Strength: Weak Recommendation).
  c) ED physicians who elect to observe infants younger than 3 months at PECARN intermediate risk of ciTBI following a minor head trauma should consider to observe them for 24 h. (Evidence Quality: D; Recommendation Strength: Weak Recommendation).
  d) Children who require observation in the ED following a head trauma should be appropriately monitored by clinical staff who are qualified to deliver care to children. (Evidence Quality: D; Recommendation Strength: Weak Recommendation).
  e) ED physicians should not repeat a CT scan and/or hospitalize solely for neurologic observation previously healthy children without intracranial injury on initial head CT, unless persistent symptoms or clinical deterioration occur. (Evidence Quality: A; Recommendation Strength: Strong Recommendation).

#### Neurosurgical consult

13. a) In children presenting to the ED following a minor head trauma and with a personal history of neurosurgical intervention other than isolated placement of a ventricular shunt, clinicians may require a neurosurgical consult, considering the type and time of the intervention, to help support CT-scan decision making. (Evidence Quality: D; Recommendation Strength: Weak Recommendation)
  b) ED physicians must discuss with a neurosurgeon the care of all children with traumatic injuries on CT scan, (excluding uncomplicated isolated linear skull fractures). For children presenting with severe head trauma ED physicians should alert a neurosurgeon as soon as possible, ideally prior to CT scan performance. (Evidence Quality: X; Recommendation Strength: Strong Recommendation).

### Inter-hospital transfer

#### Centers without CT scan

14. a) ED physicians working in centers with no CT availability should transfer all children presenting with head trauma and either a GCS < 14 or at PECARN high risk for ciTBI to referral pediatric centers with neurosurgical capability. (Evidence Quality: A; Recommendation Strength: Strong Recommendation)
  b) ED physicians working in centers with no CT availability should consider to transfer children at PECARN intermediate risk for ciTBI to referral pediatric centers, preferably with pediatric neurosurgical capability. Decision to transfer should take into consideration the availability of resources for appropriate clinical monitoring, the age of the child (transfer should be preferred in children <3 months) and physician experience. (Evidence Quality: D; Recommendation Strength: Weak Recommendation).

#### Centers with CT scan but without neurosurgery unit

15. a) ED physicians working in centers with CT capability but without neurosurgery must follow local healthcare system network guidelines for decision-making on transfer of children with moderate-severe head trauma to referral centers. Each regional system needs to have guidelines and protocols in place to ensure safe, timely and appropriate inter-hospital transfer of these children. (Evidence Quality: X; Recommendation Strength: Strong Recommendation)
  b) In centers with CT availability, but without neurosurgery, ED physicians may perform a head CT scan of children with moderate-severe head trauma, after stabilization, only if it does not delay transfer to the definitive care referral center and provided that images are of good quality and can easily be transferred to the referral center. (Evidence Quality: D; Recommendation Strength: Weak Recommendation).
  c) In centers with CT availability but without neurosurgery children with minor head trauma should be managed according to the recommendations previously provided in these guidelines for CT scan decision-making (KAS 6) and request of neurosurgical consultation (KAS 13). ED physicians should use teleradiology, whenever available, to discuss with the referral neurosurgical unit the transfer of children with traumatic brain inury on CT. (Evidence Quality: B; Recommendation Strength: Strong Recommendation).
  d) ED physicians working in centers with CT capability but without neurosurgery should transfer to referral pediatric centers children with minor head trauma who need clinical observation whenever resources for appropriate clinical observation are not available in the referring center. (Evidence Quality: X; Recommendation Strength: Strong Recommendation).
  e) ED physicians working in centers with CT capability but without neurosurgery should transfer to referral pediatric centers, preferably with pediatric neurosurgical capability, children with minor head trauma needing sedation to undergo CT scan, if no skilled staff in pediatric sedation are available at the referring center (Evidence Quality: X; Recommendation Strength: Strong Recommendation).

#### Discharge from the ED

16. a) ED physicians should ensure the following criteria are met before previously-healthy children with head trauma are discharged from the ED, either after initial assessment or following a period of observation:
  - GCS 15
  - Asymptomatic or significant improvement in symptoms
  - Normal neurological exam
  - No suspicion of child abuse
  - Reliable caregivers and ability to easily return to the ED
  - No other injuries requiring admission

For children who have undergone a head CT scan

g. Normal findings or presence of isolated linear skull fracture
h. Minor intracranial injuries on CT, based on neurosurgical consultation

(Evidence Quality: X/A; Recommendation Strength: Strong Recommendation)

- b) ED physicians should give verbal and printed discharge advice to children with head trauma and their caregivers upon discharge from the ED or ED observation unit.

The advice given should include:

- Signs and symptoms that warrant medical review
- The recommendation that a responsible adult should monitor the patient for the first 24 h after trauma
- Details about the possibility of persistent or delayed symptoms following head trauma and whom to contact if they experience ongoing symptoms
- Information about return to school and return to sports for children who sustain a concussion

(Evidence Quality: B; Recommendation Strength: Strong Recommendation)

## Background

In developed countries, injury is the leading cause of death and disability in children, with head injury being the most common type of injury [1, 2] and one of the most common reasons for presentation to the emergency department (ED) [3]. Between 600,000 and 700,000 children are evaluated annually in the EDs in the United States for blunt head trauma, with an increasing trend over time [4, 5]. The vast majority (> 95%) of these injuries, however, are mild in severity [2].

For the small minority of children with severe head trauma clinical management has to focus on the rapid and effective physiologic stabilization of the patient in order to avoid secondary brain injury, followed by the prompt identification of intracranial injuries that may benefit from neurosurgical intervention and/or neuroprotective strategies [6].

For the large number of children with mild severity head trauma the initial assessment and management in the ED is aimed at an early identification of intracranial injuries that may lead to a poor neurologic outcome if not promptly recognized [3].

In 2002 the Italian Society of Pediatric Emergency Medicine (SIMEUP) published revised guidelines in Italian on the initial assessment and management of children presenting to the ED with head trauma [7]. Since then a significant body of literature has been published on this topic. Single institution protocols and clinical practice guidelines have been individually updated to reflect the new available evidence, leading to management heterogeneity across the country. Reducing variation in practice through use of national guidelines can help optimize resource utilization while ensuring optimal patient care.

The goal of these guidelines is to assist physicians’ decision making with an up-to-date evidence-based guidance to the assessment and management of children younger than 16 years of age who present to the ED following a blunt head trauma. The guidelines are intended for pediatric and adult physicians who care for children presenting to the ED within the first 24 h of their injury.

This document will not specifically address the assessment and management of cervical spine injuries that may be associated with head trauma.

The management of children with head trauma and a history of bleeding disorder will be addressed on a separate supplement to these guidelines.

The guidelines are not intended as a sole source of guidance in the assessment and management of children with head trauma in the ED.

These guidelines evaluates published literature following an evidence-based approach to guidelines development in order to provide evidence-based key action statements [8].

## Methods

In April 2013, SIMEUP convened a new subcommittee to develop these guidelines. The guidelines development group (GDG) included four paediatric emergency physicians, a paediatrician, a paediatric intensivist, a neurosurgeon, a neuroradiologist, a pediatric nurse and a parent representative.

All panel members were given an opportunity to declare any potential conflicts. All authors had no conflicts of interest to disclose. Participation to the guidelines process was voluntary and not paid. Travel assistance was provided by SIMEUP.

The search for evidence included guidelines as well as primary and secondary medical literature.

The following databases were searched for recent relevant guidelines on the topic:

- National Guideline Clearinghouse (www.guideline.gov)
- National Institute for Health and Care Excellence (NICE) (www.nice.org.uk)
- Scottish Intercollegiate Guidelines Network (SIGN) (www.sign.ac.uk)
- New Zealand Guideline Group (NZGG) (https://www.health.govt.nz/publications/)
- Australian National Health and Medical Research Council (www.nhmrc.gov.au)
- Italian Health Guideline Database (Sistema Nazionale Linee Guida –SNLG) (www.snlg-iss.it)

The search of primary and secondary medical literature was conducted on the PubMed and The Cochrane Library electronic databases. Both MeSH terms and free text were used in order to maximize the sensitivity of the search strategy and include the most recent articles that had not undergone MeSH indexing at the time of the search. Further adjustments were implemented following the feedback of content area experts who were best able to identify known relevant articles that were missed throughout the original searches. All searches were limited to English-language and pediatric age (birth to 18 years). Relevant papers were selected among systematic reviews or meta-analysis, randomized controlled trials, observational studies, case series and case reports where appropriate. Narrative reviews, editorials and letters to the editors were excluded. After a first selection based on title/abstract, relevant papers were identified by reading the full-text. Results were supplemented with literature identified from reference lists or recommended by peers. Studies were included if focusing on a population younger than 16 years or if the upper age limit exceeded 16 but the majority of patients were younger than 16 years. The search strategies used for each section are reported in the online supplementary Appendix.

By decision of the GDG, in taking into account the available resources, the literature review aimed at identifying an evidence base for most of the guideline recommendations encompassing the period from 15 February 2005 to 15 February 2015. The results of the systematic literature review of selected guidelines were consulted to identify relevant papers published prior to our search strategy date limit, as reported in the text. Any studies added to the databases after the strategy end date were not included unless specifically stated in the text.

**Fig. 1:**
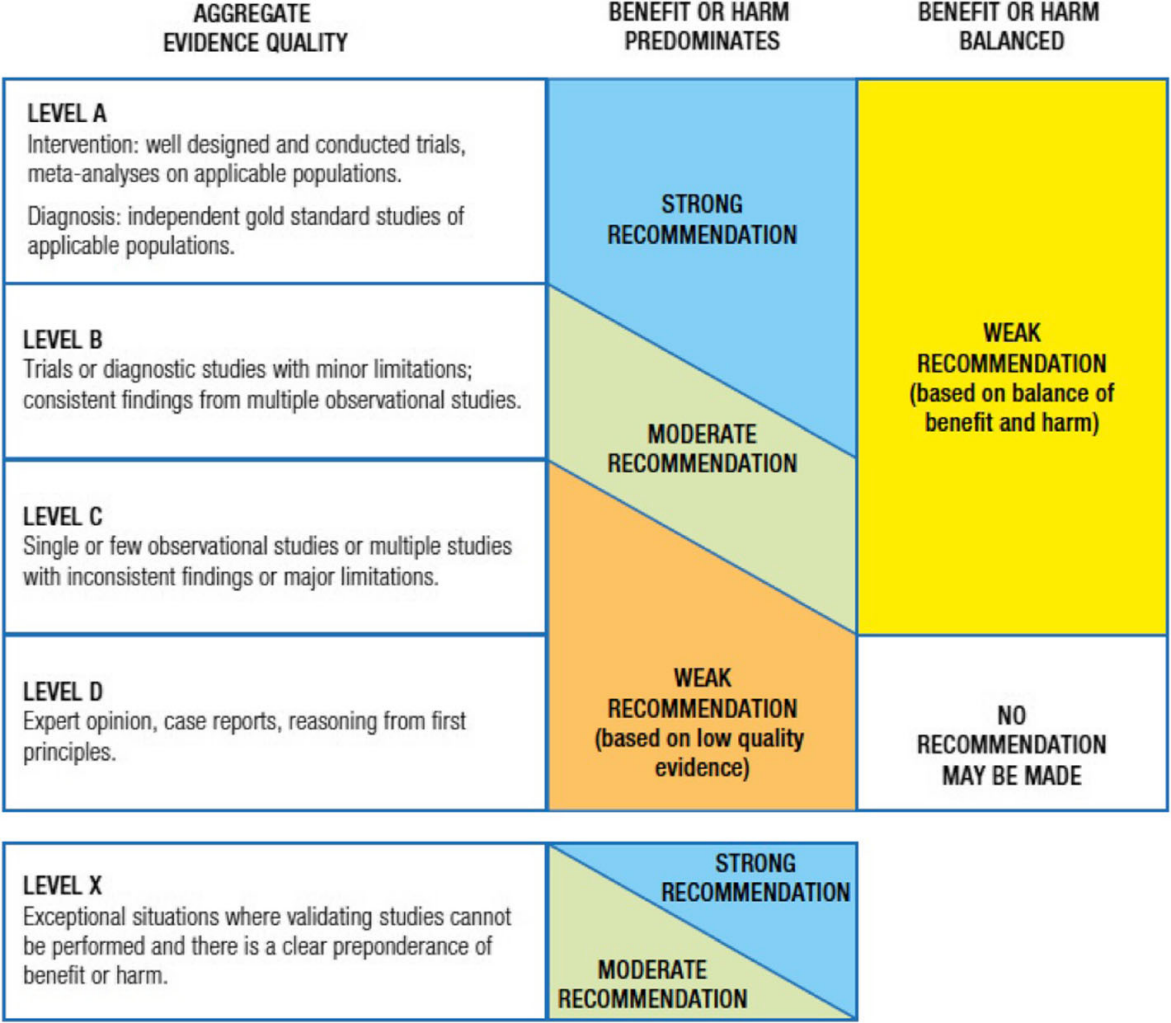
Relationship of evidence quality and benefit-harm balance in determining the level of recommendation-[8]. Rec, recommendation

The GDG followed the American Academy of Pediatrics policy statement “Classifying Recommendations for Clinical Practice” [8] in designating levels of recommendations (Fig. 1). Each key action statement indicates level of evidence, benefit-harm relationship, and strength of recommendation.

When evidence was of poor quality, conflicting or absent, the GDG drafted recommendations based on a combination of evidence and expert consensus. The considerations for making consensus based recommendations included the balance between potential harms and benefits, economic or cost implications compared to the benefits, current practices, recommendations made in other relevant guidelines, patient preferences when appropriate, and equality issues. The consensus recommendations were developed through discussions within the GDG.

The GDG formulated recommendations in three main areas of direct interest, which are presented in the text in the order in which a clinician would use them when evaluating and managing a child with blunt head trauma in the ED. These areas include initial assessment and stabilization, diagnosis of clinically important traumatic brain injury, management and disposition in the ED. The assessment and management of children with bleeding disorders will be presented separately as a supplement to these guidelines.

Coauthors drafted manuscripts for each area. The entire team gathered on a regular basis to discuss the literature base and edit the recommendations. Manuscripts were revised. Virtual meetings were held with a subset of the coauthors to complete the editing process.

The draft version of these guidelines underwent peer review by representatives of the Italian Society of Pediatric Hospitalist (SIPO), the Italian Society of Emergency Medicine (SIMEU), the Italian Society of Neurosurgery (SINch), the Italian Society of Neonatal and Pediatric Resuscitation and Anesthesia (SARNePI), the Italian Society of Neuroradiology (AINR). Comments were reviewed by the GDG and incorporated into the guidelines as appropriate.

It is important to note that the recommendations included in these guidelines are not meant to replace clinical judgment and may not provide the only appropriate approach to the management of children presenting to the ED following a blunt head trauma. Physicians need to use clinical judgment, knowledge and expertise when deciding whether it is appropriate to apply guidelines.

The working group aims to review and update these guidelines in 5 years.

### Definitions of terms used in these guidelines

- *Head trauma severity*: defined according to the initial GCS score on presentation to the ED; mild [GCS score 14–15]; moderate [GCS score 9–13]; and severe [GCS score ≤ 8] [2]; in the absence of a universally accepted definition of head trauma severity the GDG decided to use an operational definition based on initial GCS. This definition, previously used in the literature [2, 3], was deemed to be the most appropriate for the purpose of providing recommendations on the acute management of head trauma in the ED.
- *clinically important Traumatic Brain Injury (ciTBI)* [3]: defined by any of the following descriptions: death from traumatic brain injury; neurosurgical intervention for traumatic brain injury; intubation of more than 24 h for traumatic brain injury; hospital admission of 2 nights or more for ongoing symptoms or signs related to the traumatic brain injury in association with traumatic brain injury on computed tomography (CT);
- *Concussion*: according to the 2012 Zurich Consensus Statement on Concussion in Sport [9] it is defined as a complex pathophysiological process affecting the brain, induced by biomechanical forces, that may be caused either by a direct blow to the head, face, neck or elsewhere on the body with an ‘impulsive’ force transmitted to the head. It typically results in the rapid onset of short-lived impairment of neurological function that resolves spontaneously. However, in some cases, symptoms and signs may evolve over a number of minutes to hours. Concussion may result in neuropathological changes, but the acute clinical symptoms largely reflect a functional disturbance rather than a structural injury and, as such, no abnormality is seen on standard structural neuroimaging studies. Concussion results in a graded set of clinical symptoms that may or may not involve loss of consciousness. Resolution of the clinical and cognitive symptoms typically follows a sequential course. However, it is important to note that in some cases symptoms may be prolonged.

### Dissemination and implementation

Dissemination of these guidelines will occur by publication in the websites of relevant scientific societies, national and international journals, presentations at national and international conferences, education sessions and meetings with staff at individual institution level in order to assess the need for local adaptation. The implementation of these guidelines is important to help optimize the balance between patient outcome and use of resources in children presenting with blunt head trauma to the ED.

## Recommendations

### Initial assessment and stabilization

#### Key action statement (KAS) 1

Clinicians must follow the ABCDE approach according to the ATLS/PALS/EPALS principles for the initial assessment and management of all children with severe head trauma.

#### Action statement profile: KAS 1

**Table:** 

Aggregate evidence quality	X
Benefits	Improvement in survival (by treating time-critical life-threatening injuries first) and neurological outcome (by reducing secondary brain injury related to hypoxia/hypercapnia and hypotension)
Risk, harm, cost	None
Benefit-harm assessment	Benefits overweigh harms
Values judgments	None
Intentional vagueness	None
Role of patient preference	None
Exclusion	None
Strength	Strong recommendation
Difference of opinion	None

### Accompanying text

The purpose of this statement is to offer guidance on the initial assessment and management of children presenting to the ED with severe head trauma.

Since 1978, the year of the first Advanced Trauma Life Support (ATLS®) course, its systematic and structured approach has been accepted worldwide as a standard for the emergency care of trauma patients [6, 10, 11]. The assumption underlying this approach is that appropriate and timely care can significantly improve the outcome of these patients. Sequential priorities of assessment and treatment are set according to the time frames in which injury kills, emphasizing the importance of treating the greatest threat to life first. The loss of an airway kills more quickly than does the loss of the ability to breath, which in turn kills more quickly than the loss of circulating blood volume, while the presence of an expanding traumatic intracranial hematoma is the next more lethal problem. A quick but comprehensive identification of all time-critical killers also includes a thorough inspection of the patient, whose body needs to be completely exposed (undressed) for examination. Thus the mnemonic ABCDE of the primary survey defines the specific ordered assessments and interventions that should be followed in all injured patients: Airway with cervical spine protection; Breathing; Circulation (hemorrhage control); Disability (neurologic status); Exposure (undress) and Environment (temperature control). In the modern trauma team approach the ABCDE provides the mind frame to set priorities, while assessments and interventions are performed simultaneously by multiple professionals with specific allocated roles.

The ABCD approach is essential to provide timely treatment of hypoxia and hypotension in order to prevent ischemia-related secondary brain injury in patients with traumatic brain injury. In the absence of coexisting penetrating injuries blood pressure should be maintained at normal age-based values to ensure good cerebral perfusion.

Assessment of the neurologic status (D) includes a careful evaluation of the patient’s level of consciousness using the Glasgow Coma Scale (GCS), the assessment of pupillary size and reaction, lateralizing signs and spinal cord injury level. The GCS, with its pediatric version for preverbal children (Supplementary online Appendix), is predictive of patient outcome, particularly the best motor response [12]. The total GCS and its eye, vocal and motor components, as well as pupillary size and reaction should be carefully documented in the clinical notes at each assessment in order to promptly identify any neurological deterioration or improvement. Mental status may also be affected by alcohol or drug intake, as well as hypoglycemia, which should be treated immediately to prevent further damage. Blood glucose level should be checked in all patients with severe head trauma.

The initial assessment and management of patients with severe head trauma according to the ATLS principles is based on physiologic stabilization rather than treating a definitive diagnosis. A detailed history is not essential to begin the evaluation of a patient with acute injuries and focused information should be collected on patient’s *a*llergy, *m*edications, relevant *p*ast medical history, *l*ast meal and characteristics of the traumatic *e*vent (AMPLE). The secondary survey is not performed until the child has been stabilized and life-threatening conditions identified and treated. This entails a thorough systemic examination of the entire body (including log roll, a maneuver used to move a supine trauma patient on one side to examine the back for potential injuries without flexing the spinal column) to identify other present injuries and a more detailed history if possible. After the log roll, in order to avoid jugular compression and promote adequate drainage of cerebrospinal fluid (to minimize rises in intracranial pressure) the head of bed should be elevated of 30 degrees and the head and neck maintained in the neutral midline position. The cervical collar fit should be checked as a too tight collar can obstruct venous drainage.

Despite the global acceptance of the ATLS principles as gold standard in trauma management, there are few data suggesting that ATLS training has significantly reduced trauma-related morbidity and mortality in developed countries [13, 14]. A recent Cochrane review [15] found no RCT, controlled trials or controlled prospective before-after studies comparing the impact of ATLS-trained hospital staff versus non ATLS-trained hospital staff on injury mortality and morbidity. However, as the authors of the review highlight, these results are not entirely unexpected. The complexity of factors influencing trauma patient care and the difficulty to separately evaluating the impact of an education approach, such as ATLS training, from process approaches, initiatives that are entirely hospital or system-based, or experience, related to higher patient volumes, is methodologically challenging.

Given the worldwide implementation of ATLS principles as standard of care based on the biological plausibility of their benefits, high quality controlled studies to assess ATLS impact on clinically relevant outcomes in developed countries are very unlikely to be conducted in the future.

In modern trauma centers in high-income countries the appropriate identification and effective treatment of time-critical life-threatening injuries is a team effort, as multiple health care professionals with different skills sets can provide care simultaneously under the guidance of a team leader [16]. In this context the ABCDE approach provides the structure for the coordination of the multisystem assessment performed by several clinicians rather than being used a strict sequential approach to be followed in a single provider trauma scenario.

In Italy the organization of formal trauma management systems is heterogeneous and fragmented, especially for pediatric trauma [17]. While recognizing the need for a more structured and standardized trauma management system and network for pediatric patients throughout the whole country, the GDG encourages each institution serving as referral center for pediatric trauma to adopt internal guidelines and protocols to optimize the management of these patients, by tailoring the clinical management to the resources and skills available within that institution. These institutions are expected to ensure ready access to experts in the management of critically-ill children, including pediatric or adult emergency physicians, intensive care and surgical specialists, as well as radiologists for rapid reporting of acute imaging. Providers involved in acute pediatric trauma care should receive specific training, including training on crisis resource management. This focuses on non-technical skills that are essential for effective teamwork in emergency situations (e.g. leadership and followership, call for help, best use of available resources, effective communication strategies).

The GDG also encourages each institution to organize internal trauma team training to optimize coordination of care of children with severe head trauma in the ED and to facilitate transition of care to the most appropriate inpatient specialty team [18]. Both video analysis of real ED trauma resuscitations as well as high fidelity simulation have been used for this purpose [19–23]. Which is the most effective way of providing trauma team training has yet to be determined. Both education approaches have the purpose to address non-technical skills, as well as reinforcing appropriate use of available internal resources according to local policies and procedures, thus facilitating the transition from a team of experts into an expert team [24].

### Key action statement 2

In children presenting to the ED with severe blunt head trauma and signs of raised intracranial pressure (ICP) administration of hyperosmolar therapy with *hypertonic saline* should be considered.

#### Action statement profile: KAS 2

**Table:** 

Aggregate evidence quality	B
Benefits	Reduction of intracranial pressure in patients at risk of cerebral herniation following severe head trauma
Risk, harm, cost	Possible side effects of 3% hypertonic saline very unlikely following administration of one dose in the ED
Benefit-harm assessment	Under these circumstances, any adverse effects are most likely to be outweighed by therapeutic benefit
Values judgments	In making this recommendation the GDG also considered evidence from adult studies
Intentional vagueness	None
Role of patient preference	None
Exclusion	Patients with no signs of raised ICP
Strength	Moderate recommendation
Difference of opinion	None

### Accompanying text

The purpose of this statement is to offer guidance on the administration of intravenous hyperosmolar therapy in children presenting to the ED with severe head trauma and signs of raised ICP. Administration of hyperosmolar fluid to both lower blood viscosity and decrease intracerebral edema is amongst the most commonly used therapeutic options to decrease ICP in order to prevent brain herniation syndromes and secondary brain insult [25].

In the ED setting hyperosmolar therapy is used as a temporary measure to optimize patient stabilization for the transition towards either surgical treatment or neuro-protective intensive care.

There are no studies assessing the administration of hyperosmolar therapy in the ED setting neither in adult nor in paediatric patients with severe head trauma. Until such studies are available the body of evidence obtained in neuro-intensive care units should be used to guide best practice in the ED.

In 2012 Kochanek et al. [26] published the second edition of the guidelines for the acute medical management of severe traumatic brain injury (TBI) in infants, children and adolescents. The authors recommended that 3% hypertonic saline should be considered for the treatment of severe pediatric TBI associated with intracranial hypertension, at doses between 6.5 and 10 ml/kg (level II recommendation). No evidence-based recommendation on the use of mannitol could be made, as there were no pediatric studies meeting inclusion criteria. The literature search of these guidelines was updated to 2010. Since then no relevant new studies on either hypertonic saline or mannitol could be identified throughout our search strategy.

The body of evidence supporting the recommendation on hypertonic saline in children with severe TBI is based on three studies (two randomized controlled trials and one retrospective study) published between 15 and 23 years ago [27–29]. Despite the numerous limitations and differences between studies, they consistently found a positive effect of hypertonic saline in reducing ICP, while survival and length of hospital stay did not seem to be affected [26].

Adult guidelines [30] published in 2007 recommend mannitol as the hyperosmolar therapy to control raised ICP at doses of 0.25–1 g/kg in patients with severe TBI. Mannitol has been used since 1960s to reduce ICP in patients with severe TBI. However, this consolidated practice has not been supported by evidence. A recent Cochrane review concluded that there is insufficient reliable evidence to recommend the use of mannitol in the management of patients with TBI with respect to relevant clinical outcomes, i.e. death and neurologic recovery [31]. Mannitol, a dehydrating osmotic agent, can cause clinically important adverse effects, such as renal failure and hypovolemia. In the context of multiple injuries with possible concurrent bleeding or neurogenic vasodilation, mannitol-induced hypovolemia contributes to worsening hypotension, which in turn is responsible for secondary brain injury due to reduced cerebral perfusion [32]. Concerns with its use have led to interest in volume-expanding hypertonic solutions, which maintain cerebral blood flow. Their use in clinical practice was described since the 1990s [25]. To date, however, only few studies have directly compared the two agents in adults and none has assessed their administration in the ED setting. More recently various meta-analyses have summarized the results of these trials [31, 33–36]. Despite their methodological differences they all highlighted a trend favoring hypertonic saline as a more effective agent in lowering ICP. However, the lack of well-designed and sufficiently powered studies directly comparing the two agents does not definitely prove the superiority of hypertonic saline. In addition it should be mentioned that successful control of ICP does not guarantee a good neurologic outcome.

Unfortunately, dosing, concentration and duration of administration of hypertonic saline vary widely among institutions and no standard treatment protocol exists. In the ED setting we advise for administration of a bolus of 3% hypertonic saline according to local protocols. Decisions on the need for additional boluses or continuous infusion have to be referred to the intensive care team.

### Key action statement 3a

Clinicians should avoid hyperventilation in children presenting to the ED with signs of raised ICP following a severe head trauma.

#### Action statement profile: KAS 3a

**Table:** 

Aggregate evidence quality	C (based on low quality evidence)
Benefits	Avoidance of secondary brain injury due to reduction in cerebral blood flow caused by hypocapnia-induced vasoconstriction
Risk, harm, cost	Missing transient reduction in ICP
Benefit-harm assessment	Benefits outweigh risks
Values judgments	In making the recommendation the GDG also considered evidence from adult studies
Intentional vagueness	None
Role of patient preference	None
Exclusion	Patients with no signs of raised ICP
Strength	Moderate recommendation
Difference of opinion	None

#### Key action statement 3b

In children presenting to the ED with signs of impending cerebral herniation following severe head trauma, clinicians may consider hyperventilation (PaCO_2_ of 25–35 mmHg) as a temporary measure to rapidly reduce ICP in order to increase the patient chances of undergoing a life-saving intervention.

#### Action statement profile: KAS 3b

**Table:** 

Aggregate evidence quality	D (expert consensus)
Benefits	Rapid reduction of ICP may prevent cerebral herniation and allow for definitive surgical management
Risk, harm, cost	Reduction in cerebral blood flow and worsening of secondary ischemic injury
Benefit-harm assessment	In this selected group of patients at very high risk of death any adverse effects are most likely to be outweighed by therapeutic benefit
Values judgments	Clinical experience was used in making this judgment while recognizing that extensive data from studies are lacking
Intentional vagueness	No target PaCO_2_ range was specified, as the optimal PaCO_2_ range under these circumstances remains unclear
Role of patient preference	None
Exclusion	Patients with no signs of impending cerebral herniation
Strength	Weak recommendation
Difference of opinion	None

### Accompanying text

The purpose of these statements is to offer guidance on the use of hyperventilation in children presenting to the ED with severe head trauma.

Hyperventilation has been shown to rapidly decrease ICP and increase cerebral perfusion pressure, supporting its use in clinical practice for rapid reduction of ICP since the 1970s [26, 37, 38]. However, it has been demonstrated that induced hypocapnia causes cerebral vasoconstriction and a reduction in cerebral blood flow, which determines reduced cerebral oxygenation and brain ischemia [37, 39, 40]. According to the results of clinical studies hypocapnia seems to exacerbate cerebral injury and to worsen clinical outcomes [41].

The 2012 guidelines for the acute medical management of severe pediatric TBI [26] state that “avoidance of prophylactic severe hyperventilation to a PaCO2<30mmHg may be considered in the initial 48 h after injury. If hyperventilation is used in the management of refractory ICP, advanced neuromonitoring for evaluation of cerebral ischemia may be considered”. The body of evidence behind this level III recommendation stems from two observational studies.

One was a low quality prospective case series on 23 children [42] that found a decrease in cerebral blood flow (measured by xenon computed tomography) when PaCO_2_ was reduced with hyperventilation (regional ischemia was 28.9%, 59.4% and 73.1% during normocapnia, PaCO_2_ of 25–35 mmHg and <25 mmHg, respectively). The correlation with clinical outcome was not analyzed, despite reported frequency of outcome severity.

The second report was a retrospective trauma registry-based cohort study of 464 children mechanically ventilated following severe TBI [43]. The authors recorded the frequency of severe hypocarbia (PaCO_2_ < 30 mmHg) in the first 48 h of admission. After controlling for confounders the adjusted Odds ratio (OR) for mortality was of 1.44 (95% CI 0.56–3.73) for one episode of severe hypocarbia, 4.18 (95% CI 1.58–11.03) for two episodes and 3.93 (95% CI 1.61–9.62) for three or more episodes, compared with patients with mild or no hypocarbia recorded.

Our search strategy could identify only one new relevant observational study assessing clinical outcomes of hyperventilation in children with severe TBI in addition to the studies selected for the 2012 guidelines [26].

The study by Ramaiah et al., [44] included a retrospective cohort of 194 children with severe TBI. They found that children with normocarbia (PaCO_2_ between 36 and 45 mmHg) at the time of ED admission had greater discharge survival compared to those with both admission hypocarbia (PaCO_2_ ≤ 35 mmHg) and hypercarbia (PaCO_2_ ≥ 46 mmHg). PaO_2_ 301–500 mmHg and normocapnia on admission to the ED were independently associated with discharge survival (adjusted OR 8.02, 95% CI 1.73–37.10, and 5.47, 95% CI 1.30–23.07 respectively).

Common limitations to the two larger studies [43, 44] are: the retrospective design, the inability to fully adjust for the effect of potential confounding clinical variables/events playing a role before or after PaCO_2_ measurement, the inability to evaluate the effect of duration of PaCO_2_ alterations on the study outcome and the lack of assessment of long-term outcomes.

The only high-quality RCT comparing hyperventilation versus normoventilation in patients with severe TBI was carried out more than 20 years ago in a mostly adult population [30, 39, 45]. While patients had to be aged three years or older to be included in the study, the mean age was 32 ± 18 years in the hypocapnia group (41 patients) and 28 ± 15 years in the normocapnia group (36 patients). The number of children included was not reported. This study showed that prophylactic use of sustained hyperventilation (PaCO_2_ 24–28 mmHg) for a period of 5 days retards recovery from severe TBI, with outcome being statistically significantly worse at 3 and 6 months but not at 12 months. This RCT was not double blind, and randomization was compromised early in the study, because people whose informed consent could not be obtained were assigned to the control group. This practice was ceased as soon as the authors became aware of it.

A recent systematic review [41] including patients of all ages with acute cerebral injury found evidence suggesting that hypocapnia and hypercapnia are associated with increased rates of poor outcome both overall and in the severe TBI subgroup. High quality clinical trials, however, are lacking and the optimal PaCO_2_ range as well as the therapeutic time window during which optimization of PaCO_2_ has the greatest impact remain unclear.

No pediatric studies have thus far specifically assessed the effects of varying levels of hypocapnia on ICP or outcome, or have evaluated the transient use of hyperventilation in the setting of impending herniation.

In view of the lack of evidence to guide ED physicians on the use of hyperventilation in children with severe TBI the GDG issued a consensus-based recommendation targeting patients who could benefit the most from rapid reduction of ICP induced by hyperventilation. In the ED setting rapid control of ICP may be especially useful as a short-term temporary measure for patients with signs of impending cerebral herniation who are amenable of life-saving neurosurgical hematoma evacuation. In this context, rapid reduction of ICP by hyperventilation has the purpose to maximize the patients’ chances of undergoing a life-saving surgical operation before cerebral herniation occurs.

Future high-quality clinical trials of PaCO_2_ management in the ED should focus on the assessment of varying PaCO_2_ ranges to allow for identification of the optimal PaCO_2_ target and therapeutic time window. Such studies should be conducted in homogeneous populations of children with severe TBI, including children with impending cerebral herniation and intracranial lesions amenable of life-saving neurosurgical interventions. Until further evidence is available the consensus from the GDG recommends a PaCO2 target between 35 and 40 mmHg in head injured children with clinical signs of raised ICP, but no impending cerebral herniation.

### Key action statement 4

In children presenting to the ED with severe blunt head trauma steroids should not be administered.

#### Action statement profile: KAS 4

**Table:** 

Aggregate evidence quality	B
Benefits	Avoidance of potential risk for mortality; suppression of endogenous free cortisol levels; possible higher risk of bacterial infections
Risk, harm, cost	None
Benefit-harm assessment	Benefits outweigh harms
Values judgments	In making the recommendation the GDG also considered evidence from adult studies
Intentional vagueness	None
Role of patient preference	None
Exclusion	Patients with mild-moderate TBI
Strength	Strong recommendation
Difference of opinion	None

### Accompanying text

The purpose of this statement is to offer guidance on the administration of steroids in children presenting to the ED with severe head trauma.

The positive effects of corticosteroids in reducing edema and improving outcome in patients with brain tumors led to their use as neuroprotective agents in patients with severe TBI. Two adult trials from the 1970’s showed a favorable effect of steroid administration on the outcome of patients with severe TBI [46, 47]. However, subsequent studies in adults with TBI not only failed to show any beneficial effects of steroid use on clinical outcome or ICP reduction, but highlighted the potential for increased mortality [30, 48].

The 2012 guidelines for the acute medical management of severe TBI in infants, children and adolescents [26] state that steroids are not recommended to improve clinical outcome or reduce ICP (level II recommendation). The literature search of these guidelines was updated to 2010. Since then no relevant new studies on the use of steroids in children with TBI could be identified throughout our search strategy.

The body of evidence meeting the 2012 guidelines inclusion criteria to support the recommendation on steroids [26] is based on two reports of the same trial [49, 50]. This randomized placebo-controlled trial included 25 patients, age range between 1.4 and 15.8 years, with a GCS ≤ 7. Thirteen patients received dexamethasone at a dose of 1 mg/kg/day for 3 days. Dexamethasone had no effect on either ICP reduction or Glasgow Outcome Scale at 6 months. The authors found a significant suppression of endogenous cortisol and a trend towards increased bacterial pneumonia in the dexamethasone group.

The same study was excluded from a Cochrane review on corticosteroids for acute traumatic brain injury, last updated in 2009, [48] because the allocation concealment was reputed inadequate after contact with the authors. This review identified three other pediatric studies. One was excluded because retrospective [51]. Of the two included studies, one was published in Spanish and had a total population of only 10 patients [52], while the other included only patients with a GCS ≥ 9 [53]. Data of included studies were not pooled because of significant heterogeneity. The largest included trial [54, 55] on over 10.000 adult patients shows a significant increase in death with steroids (risk ratio of 1.18, 95% CI 1.09 to 1.27), and raises serious concerns on the use of steroids for both adult and pediatric patients with severe TBI.

### Key action statement 5

In children presenting with severe blunt head trauma hypothermia should not be initiated in the ED.

#### Action statement profile: KAS 5

**Table:** 

Aggregate evidence quality	A
Benefits	Avoidance of potential for increased mortality
Risk, harm, cost	None
Benefit-harm assessment	Benefits outweigh harms
Values judgments	None
Intentional vagueness	None
Role of patient preference	None
Exclusion	Patients with mild and moderate TBI
Strength	Strong recommendation
Difference of opinion	None

### Accompanying text

The purpose of this statement is to offer guidance on initiation of hypothermia in the ED for children presenting with severe TBI.

Pre-clinical and clinical brain injury studies have shown that hypothermia decreases the acute pathophysiologic response and secondary damage and may reduce ICP [56–61]. These results seeded hope for its potential to improve clinically relevant outcomes, such as long-term functional neurological outcomes and mortality.

No pediatric studies have evaluated initiation of hypothermia in the ED setting. This likely reflects logistical, clinical and research challenges in the ED setting and in the transition of care to the intensive care unit (ICU). The body of evidence supporting this recommendation was obtained from studies where patients were either enrolled in the ED or ICU, but hypothermia was initiated in ICU.

While the 2012 guidelines [26] advised for consideration of moderate hypothermia (32–33 C) beginning within 8 h after severe TBI for up to 48 h duration to reduce ICP (level II recommendation), the most recent evidence does not support the use of hypothermia in children with severe TBI [62–65].

Four randomized controlled trials (RCT) assessing clinically relevant outcomes on significant pediatric samples constitute the basis for the present recommendation [62–65]. All these studies were published in the last decade.

The first prospective multicenter phase II RCT by Adelson et al. [62] in 75 children with severe TBI (GCS 3–8) determined the safety and performance of surface-induced moderate hypothermia (32–33 °C), and provided the efficacy data to support larger phase III trials. In this trial the duration of moderate hypothermia was 48 h and rewarming occurred at a rate of 0.5–1 °C every 3–4 h. Hypothermia was felt to be safe due to lack of increased mortality and no difference in complications compared with normothermia. This study also showed a potential for reduced mortality and improved outcomes in a subgroup analysis of patients with a post-resuscitation GCS of 4–8, who received hypothermia within 6 h of injury [62].

A phase III multicenter international (Canada and Europe) RCT of moderate hypothermia (32–33 °C) for 24 h, initiated within 8 h (but with an actual mean initiation of cooling of 6.3 h, range of 1.6–19.7 h) of severe TBI in 225 children and adolescents, reported that hypothermia worsened outcomes at 6 months post-injury and possibly increased mortality (21% vs 14%; *p* = 0.06) [63]. However, issues related to study design and characteristics of patients allocated to the hypothermia group raised concerns about the study findings [56, 66].

Based on preliminary work and issues from previous studies, a subsequent phase III RCT was designed to ensure early randomization and initiation of cooling, longer cooling periods, slower rewarming, and strict protocols for management of patients compared with previous pediatric severe TBI hypothermia trials [64]. After enrollment of 77 patients this trial was stopped due to futility because hypothermia, initiated within 6 h from injury and used globally for 48–72 h, followed by a slow rewarming period, did not improve mortality or global function 3 months after injury compared with normothermia.

These three trials have been included in a recent meta-analysis along with other four smaller RCTs of lower methodological quality [67]. The results of the meta-analysis showed that mortality rate was higher in the hypothermia group compared with the normothermia group. This result was stable and statistically significant even after change in the statistical model and effect size and removal of confounding factors. When considering only studies with higher methodological quality, the relative risk for mortality was 1.75, (95% CI 1.06–2.90, *p* = 0.03). In addition neurological outcome measured by the Glasgow Outcome Scale at 3 and 6 months was overall not significantly different between the two groups, while a significantly higher incidence of arrhythmias was found in the hypothermia group (RR 2.60, 95% CI 1.06–6.41, *p* = 0.04) with a low statistical heterogeneity.

The most recently published study, not included in the meta-analysis, was a phase II multicenter RCT involving all Australia and New Zealand pediatric ICUs as well as one Canadian ICU [65]. Fifty patients were randomized to either moderate hypothermia (32–33 °C) for at least 72 h, initiated within 6 h of injury with rewarming rate guided by ICP and cerebral perfusion pressure, or normothermia. The investigators found no difference between the groups with respect to adverse events, neurologic outcome (pediatric cerebral performance category at 12 months) and mortality.

Further research might be beneficial to assess the potential therapeutic role of hypothermia in severe pediatric TBI under other circumstances. Future clinical trials will likely benefit from improved stratification by injury (to account for the considerable heterogeneity of lesion types and prognoses in TBI patients), alternative outcome measures, as well as alternative approaches to trial design.

## Diagnosis of clinically important traumatic brain injury

### CT scan decision-making

#### Key action statement 6a

Physicians should perform a head CT in all head injured children presenting to the ED with a GCS < 14.

#### Key action statement 6b

**Fig. 2:**
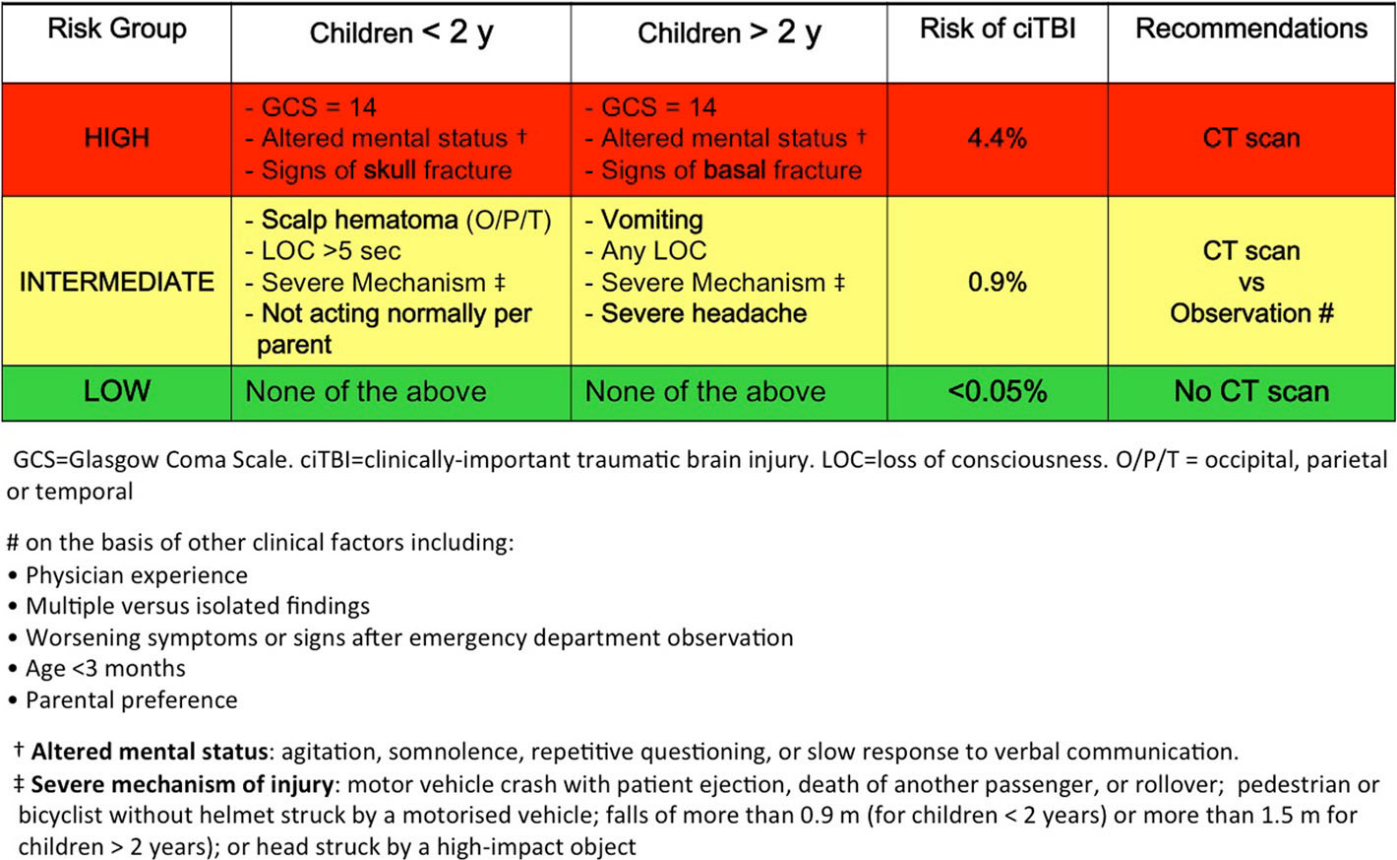
PECARN algorithms for the emergency department management of minor head trauma – Adapted from [3]

Physicians should use the age-appropriate PECARN algorithms (Fig. 2) to assist their decision-making about head CT scan in children with a GCS ≥ 14.

#### Key action statement 6c

Physicians should favor initial observation over CT scan for children at intermediate-risk for clinically important traumatic brain injury (ciTBI) according to the age-appropriate PECARN algorithms, especially in the presence of isolated findings.

#### Action statements profile, KAS 6a, 6b, 6c

**Table:** 

Aggregate evidence quality	A
Benefits	Limitation of exposure to risks related to radiation and possible need for sedation, as well as reduction in costs, for children at negligible risk of ciTBI
Risk, harm, cost	Negligible risk of missing a ciTBI. Costs of observation over CT scan as initial option for children with GCS ≥ 14
Benefit-harm assessment	Benefits outweigh harms
Values judgments	EDs that adopt this strategy should have internal guidelines/protocols in place for: - close monitoring of head injured children during observation (when chosen as initial option for children with GCS ≥ 14) - provision to families of discharge instructions on when to return to the ED for symptoms possibly related to the head trauma
Intentional vagueness	None
Role of patient preference	Parents preference should be considered for patients at PECARN intermediate-risk of ciTBI
Exclusion	Children with bleeding disorders, underlying neurologic risk factors and suspect child abuse
Strength	Strong recommendation
Difference of opinion	None

### Accompanying text

The purpose of these statements is to offer guidance on decision-making about whether to order a head CT scan in children with blunt head trauma presenting to the ED.

The main goal of the emergency physician when assessing a stable child with a head injury is to identify a skull or intracranial injury that requires a prompt treatment to achieve the best outcome for the patient. The gold standard for the identification of such injuries is computed tomography (CT) of the head.

However, CT scan bears long-term risks related to radiation exposure, with an estimated cancer lifetime attributable risk of cancer between 1:1000 and 1:10.000 pediatric head CTs in children younger than five years of age [68]. In addition, a small number of uncooperative children may need sedation to successfully undergo a CT scan with the potential, although quite low, risks related to sedation [69, 70]. Resource utilization and costs should also be taken into consideration when ordering a CT.

The rate of traumatic brain injury on CT scan varies with the severity of head injury, and it is estimated to be 65% for children with a GCS ≤ 8, 27% when the GCS on presentation is between 9 and 13 and approximately 5% for children with a minor head trauma (MHT), defined as a GCS of 14 or 15, who undergo a CT scan [2]. While the high risk of intracranial injuries in children with a GCS ≤ 13 justifies ordering a CT scan in these patients, children with a MHT pose the greatest challenge with respect to CT scan decision-making. For these patients, who represent >95% of head injured children [2] clinicians should accurately balance the risk of missing a potentially devastating intracranial injury versus the risks related to CT.

Several studies have highlighted an increase in CT scan trends in the Emergency Department in North America up to 2005 [68, 71] with significant variation in practice in different centers [72–74]. These data are currently not available for the Italian setting. However, compared with the North American setting, the CT rate for children presenting to the ED with a MHT is much lower in Italy [75, 76]. Nevertheless decision-making on CT in children with MHT continues to be a challenge also in the Italian setting, and appropriate selective CT use should be pursued.

In order to help clinicians support their decision-making recent research has focused on the development of clinical prediction rules (CPRs). CPRs are decision-aids that use three or more variables from history, physical examination, or simple tests to provide the probability of an outcome or suggest a diagnostic or therapeutic course of action for an individual patient [77]. They are potentially powerful tools for reducing uncertainty, variability and improving accuracy in medical decision-making by standardizing the collection and interpretation of clinical data. CPRs promote optimization of clinical management by reaching the most convenient trade-off between patient risks and benefits.

Many CPRs have been developed for MHT in children in the past 15 years. Cost-effectiveness analysis studies have shown that selective use of CT based on CPRs is more cost-effective than either the “CT all” or “CT none” strategy [78–81]. An observational study reported a higher sensitivity for the hypothetical use of a CPR compared with clinical judgment [82]. CPRs have been comprehensively described and compared by numerous reviews [80, 83–85]. A meta-analysis, however, could not be performed due to CPRs heterogeneity in their inclusion/exclusion criteria, as well as outcome definitions. While many rules used as their primary outcome an abnormal CT, the most recent CPRs were developed using patient-centered outcomes. These refer to clinically important intracranial injuries that require medical or surgical intervention, and thus have relevant implications for patient care [83–85]. The use of patient-centered outcomes overcomes the imperfect sensitivity and specificity of CT scans, allows minor or incidental findings to be ignored, and remain pertinent with newer and changing imaging techniques.

In order to be incorporated in patient care, CPRs must adhere to high methodologic standards and show a high diagnostic accuracy (ideally high sensitivity and specificity). The methodologic steps include: development (derivation), testing of the rule on a different population to test reliability and reproducibility of the rule (validation), assessing the impact of the rule on physician behavior and clinical outcomes (impact analysis) and widespread adoption of the rule in clinical practice (implementation) [86–90].

According to the hierarchy of evidence for CPRs published by the Evidence-Based Medicine Working Group [91] prediction rules that have been derived but not validated are the lowest level of evidence (level 4), rules that have been prospectively validated in only 1 sample are level 3, rules that have been broadly validated in multiple settings are level 2, and rules that have had impact analysis performed and demonstrated a change in clinician behavior with beneficial consequences are level 1.

The more recent reviews on CPRs for pediatric head injury identified three rules as the best quality rules according to their high derivation methodological standards [84, 85]. The three rules are the Children’s Head Injury Algorithm for the Prediction of Important Clinical Events from the UK (CHALICE) [92], the Canadian Assessment of Tomography for Childhood Head Injury (CATCH) from Canada [93], and the prediction rule for identification of children at very low risk of clinically important traumatic brain injury (ciTBI) developed by the Pediatric Emergency Care Applied Research Network (PECARN) from the US [3]. The PECARN rule is the only CPR that includes two separate rules: one for children younger and the other for children older than two years of age. This important distinction reflects the different age-appropriate clinical assessment, which takes into account the different developmental stage of pre-verbal children. The PECARN rules were then framed into risk-group algorithms (Fig. 2).

These rules characteristics have been compared in detail in the review by Lyttle et al. [85]. The same authors have also assessed the applicability of each rule to the general head injury population presenting to a pediatric emergency department. Applicability intimately relates to the inclusion and exclusion criteria used by each rule. They found that the CHALICE rule, which has the broader inclusion criteria can be applied to 97.2% of the patients; the PECARN rule to 75%, and the CATCH rule, which has the more selective inclusion criteria, to only 26.3% of patients [85]. This difference in the target population needs to be taken into account when considering which rule to adopt in each individual setting.

Besides their applicability, the three rules greatly vary with respect to their validation. High-quality validation studies are currently scarce. Validation of head injury CPRs for children has been identified as one of the research priorities by recent systematic reviews [77, 80] and the 2014 update of the NICE head injury guideline [94].

At present the most validated appears to be the PECARN rule, which has undergone prospective validation in large patient cohorts both internally [3] and externally [95, 96]. A retrospective validation of the PECARN rule on prospectively collected data from the CHALICE cohort has also been performed in children older than five years [97].

The CATCH rule has been prospectively validated in a large Canadian cohort [98] as well as in a smaller cohort of patients in the United States, where PECARN and CHALICE were also tested and compared [96]. However, in the latter study the validation cohort was selected according to different inclusion criteria and the outcome measures used did not exactly match the original CPRs’. Proper external validation requires the application of the specific inclusion and exclusion criteria and outcome definitions used in the original rules.

The CHALICE rule has only been prospectively validated in the above mentioned study, which compared the three rules in the same cohort of patients in the United States [96]. Other three studies retrospectively applied the CHALICE rule. One study, however, included a population of hospitalized patients, rather than seen in the ED [99]. The other two studies only calculated the rate of CT scan had the CHALICE rule been applied compared with actual practice on 496 and 1091 patients respectively [100, 101]. Both found an increase in the CT rate (from 1.7% to 10.6% in the study by Harty et al..., and from 19% to 46% in the study by Crowe et al) had CHALICE been applied, with no increase in accuracy in the identification of intracranial injuries needing neurosurgery.

Retrospective validation of CPRs bears many limitations and biases related to the study design. Only prospective validation studies have been included to support the present recommendation.

The characteristics of the prospective validation studies for each rule are reported in Table 1.

**Table 1:** Characteristics of prospective validation studies published for the highest-quality clinical prediction rules

	Setting	Population^a^	Outcome prevalence	Performance	Limitations/Comments
PECARN [3]					
Kuppermann 2009 [3]	25 PED in the US	8627 patients (2216 < 2y; 6411 > 2y) Same as original rule	ciTBI = 88 (25 < 2y; 63 > 2y)	< 2y SENS 100 (95% CI 86.3–100) SPEC 53.7 (95% CI 51.6–55.7) NPV 100 (95% CI 99.7–100) PPV 2.4 (95% CI 1.6–3.5) > 2y SENS 96.8 (95% CI 89.0–99.6) SPEC 58.2 (95% CI 57.0–59.4) NPV 99.95 (95% CI 99.80–99.99) PPV 2.2 (95% CI 1.7–2.9)	Internal validation
Schonfeld 2014 [95]	1 PED in the US; 1 PED in Italy	2428 patients (956 < 2y; 1472 > 2y) Same as original rule	ciTBI = 19 (6 < 2y; 13 > 2y) Positive CT = 69	< 2y SENS 100 (95% CI 64.3–100) SPEC 43.2 (95% CI 40–46.3) PPV 1.7 (95% CI 0.6–3.2) NPV 100 (95% CI 99.4–100) > 2y SENS 100 (95% CI 79.4–100) SPEC 48.3 (95% CI 45.8–50.9) PPV 2.0 (95% CI 1.1–3.2) NPV 100 (95% CI 99.8–100)	- Low number of study outcome (*n* = 19; NS = 0) - 17.5% of record retrospectively collected following prospective implementation of the rule - Incomplete clinical follow up
Easter 2014 [96]	1 PED in the US	1009 patients Included GCS = 13 (0.4%)	ciTBI = 21	SENS 100 (95% CI 84–100) SPEC 62 (95% CI 59–66) LR+ 2.7 (95% CI 2.5–2.9) LR- 0 (95% CI 0-undefined)	Included patients with GCS = 13 (but only 4 patients) Only overall rule performance reported
CATCH [93]					
Osmond 2012 [98]	9 PED in Canada	4060 patients Same as original rule	Injury requiring NS = 23 Acute brain injury on CT = 197	For the four high risk factors - for injuries needing NS SENS 87 (95% CI 68–98) SPEC 87 (95% CI 86–88) PPV 3.6 (95% CI 2.3–5.5) NPV 99.9 (95% CI 99.8–100) For the 7 CATCH predictors - for acute brain injury on CT SENS 98 (95% CI 95–99) SPEC 65 (95% CI 64–67) PPV 12.7 (95% CI 11.1–14.4) NPV 99.8 (95% CI 99.6–99.9)	Abstract form only. *(Complete accuracy measures calculated based on the numbers provided to the authors to the NICE head injury guideline working group – see Table 15 of the NICE guideline appendices)* Predicted CT rate: 14% for identifying injuries that require neurological intervention; 38% for acute brain injury on CT
Easter 2014 [96]	1 PED in the US	1009 patients not selected based on CATCH symptoms; presenting within 24 h	Injury requiring NS = 4 Any injury on CT = 52	For injury requiring NS SENS 75 (95% CI 19–99) SPEC 43 (95% CI 40–46) LR+ 1.3 (95% CI 0.7–2.3) LR- 0.6 (95% CI 0.1–3.2) For any injury on CT SENS 90 (95% CI 79–97) SPEC 45 (95% CI 42–48) LR+ 1.6 (95% CI 1.5–1.8) LR- 0.4 (95% CI 0.3–0.6)	Different inclusion criteria compared with original rule Outcome not exactly as in original rule
CHALICE [92]					
Easter 2014 [96]	1 PED in the US	1009 patients up to 18 years of age; presenting within 24 h	Injury requiring NS = 4 Any injury on CT = 52	For injury requiring NS SENS 75 (95% CI 19–99) SPEC 84 (95% CI 81–86) LR+ 4.5 (95% CI 2.5–8.2) LR- 0.3 (95% CI 0.1–1.6) For any injury on CT SENS 64 (95% CI 47–79) SPEC 86 (95% CI 83–88) LR+ 4.4 (95% CI 3.3–5.9) LR- 0.4 (95% CI 0.3–0.6)	Different age limit compared with orginal rule Outcomes not exactly as in original rule

ciTBI = clinically important traumatic brain injury (as per PECARN definition); LR + = positive likelihood ratio; LR- = negative likelihood ratio; NS = neurosurgery; PED = Pediatric Emergency Department; SENS = sensitivity; SPEC = specificity; US = United States

^a^difference with the original CPR population are reported where appropriate

The comparison of the three rules and physician practice in 1009 children presenting to the ED within 24 h of their injury with a GCS of 13–15 [96] showed that only PECARN and physician practice identified all the ciTBIs, with PECARN being slightly more specific.

External validation and diagnostic accuracy comparison of the three rules has recently been performed in a prospective cohort of 20.000 children from an observational multicenter Australian study [102, 103]. The sensitivity of the rules were high when externally validated as designed. However, when the three rules were applied to a comparison cohort, the PECARN rule missed the fewest patients [103].

Projected rate of cranial CT scan based on the original rules are 14.1% for CHALICE, 30.2% for CATCH using the four high risk factors and 51.9% using all the 7 CATCH rule factors [85]. In contrast to the CATCH and CHALICE rules that were developed to identify children for whom a CT scan is recommended, the PECARN rule was developed to identify children at very low risk of ciTBI, who could safely avoid CT. This group included 53.5% of patients younger than 2 years and 58.3% of older patients in the original rule population. This is not equivalent, however, to projected CT rates of 46.5% and 41.7% in the two age groups respectively. Based on the PECARN predictors the authors developed two algorithms to support CT decision-making (Fig. 2).

For children not meeting the very low risk criteria they identified a high-risk group, for whom a CT scan is recommended (14% of patients in the two age groups) and a moderate-risk group (32.6% younger than 2 years and 27.7% older than 2 years). The authors recommended practitioner discretion for obtaining CT scans in this group, citing observation as the alternative course of action. In contrast with the CATCH and CHALICE rules, which are directive in recommending a specific course of action, the PECARN algorithms are directive for identifying whom not to scan, but assist clinicians decision-making on whom to scan by empowering clinicians and parents with traumatic brain injury risk data for informed decision-making about CT use versus observation.

A before-after study recently carried out in an Italian Pediatric ED showed that the implementation of the PECARN algorithms in clinical practice did not lead to an increase in the CT rate, while achieving high medical staff satisfaction with their use. This seems related to the local culture of favoring observation over CT scan for children who are not at high risk of ciTBI [76].

Another study conducted in the same center following implementation of the PECARN algorithms showed that a CT was performed in only 13% of children at intermediate risk for ci TBI [104]. Of the 308 patients included only one (0.3%) had an initially missed ciTBI that did not need neurosurgery. In this study, being younger than 3 months of age was the only variable that was significantly associated with whether a CT scan was performed in this patient risk group. Symptom trend over time rather than the presence of isolated or multiple findings seemed to play a more important role in decision-making.

To further support decision-making for children at PECARN intermediate risk, several secondary planned analyses of the original PECARN dataset reported specific risk estimates of ciTBI for isolated moderate-risk findings (Table 2).

**Table 2:** PECARN secondary analyses results on isolated moderate-risk findings for ciTBI

Isolated moderate risk PECARN findings	Reference	Population	Outcome risks n/N, (%; 95%CI)	
			ciTBI	TBI on CT
Loss of consciousness	Lee, [163]	<2y	1/157 (0.6; 0.0–3.5)	2/90 (2.2; 0.3–7.8)
		≥2y	12/2623 (0.5; 0.2–0.8)	36/1903 (1.9; 1.3–2.6)
Severe injury mechanism	Nigrovic, [164]	<2y	4/1327 0.3 (0.1–0.8)	NA
		≥2y	12/1975 0.6 (0.3–1)	NA
Vomiting	Dayan, [137]	<2y	0/567 (0; 0–0.6)	2/187 (1.1; 0.1–3.8)
		≥2y	10/1501 (0.7; 0.3–1.2)	26/806 (3.2; 2.1–4.7)
Severe Headache	Dayan, [165]	≥2y	0/91 (0; 0–4.0)	0/50 (0; 0–7.1)
Non-frontal scalp hematoma	Dayan, [167]	<2y	12/2998 (0.4; 0.2–0.7)	50/570 (8.8; 6.6–11.4)
Not acting normally per parent	Nishijima [166]	<2 y	1/411 (0.2; 0–1.3)	4/185 (2.2; 0.6–5.4)

ciTBI definition: death, neurosurgical procedure, intubation for at least 24 h for TBI, or hospitalization for 2 or more nights because of the head trauma in association with TBI on cranial CT

TBI on CT: any acute traumatic intracranial fi nding or a skull fracture depressed by at least the width of the skull. Patients with isolated skull fractures that were not depressed by at least the width of the skull were not considered as having traumatic brain injury on CT;

*NA* not available

Cost-effectiveness analyses comparing different rules found that the most cost-effective rule was CHALICE when using derivation data [79, 80]; however, some uncertainty remains, as incremental changes in the costs and quality-adjusted life years were very small when all selective CT strategies were compared. In addition the most recent validation data were not used in these analyses.

Another cost-effectiveness analysis comparing the PECARN strategy with usual care [81] found that the former was more effective (less total quality adjusted life-year loss) and less costly than the usual care strategy. The PECARN prediction rules were associated with less frequent cranial CT use, fewer radiation-induced cancers, lower total costs, and lower total quality-adjusted life-year loss compared with a strategy based on usual care. The PECARN strategy was more cost-effective than the usual care strategy until the latter’s CT scan rate decreased to 26%.

According to a cost-effectiveness analysis including only children younger than 2 years the probability of ciTBI of approximately 5% is the threshold above which CT all becomes the preferred strategy. The threshold decreases with less radiation. As new technology allows CT scans to be carried out even faster, and possibly with a lower dose of radiation, the usefulness of CPRs for pediatric head trauma in the future may change significantly.

None of the rules has undergone actual large-scale impact analysis. The actual impact of a CPR will depend on how its predictions are translated into decisions and how clinician input is effectively incorporated before, during and after testing in actual practice. An Italian study [76] showed a high adherence to the PECARN algorithms in clinical practice, as well as a high safety and efficacy profiles. The study, however, was limited by the small number of patients included (288 patients before and 356 after the implementation of the PECARN algorithms).

In light of the high methodological quality and high diagnostic accuracy, the consistent findings of prospective validation studies, the results of the available cost-effectiveness analysis, as well as the Italian studies describing the successful implementation and use of the PECARN algorithms in clinical practice, the GDG recommends the proposed PECARN decision-making strategy to be used nation-wide for decision on CT scan in children presenting with MHT to the ED.

While awaiting the results of the large Australian multicenter study on the concurrent external validation of the PECARN, CHALICE and CATCH rules [102], the effects of the use of the PECARN algorithms in clinical practice in Italy should be closely monitored. These results, in addition to a cost-effectiveness analysis of this approach within the Italian health care system would provide comprehensive data to further support or change this recommendation for the future.

Whenever a head CT scan is necessary clinicians must ensure that radiation doses are optimized for pediatric patients. Age-related protocols should be preferred. If optimization is not possible, transfer to a pediatric center should be considered according to patient clinical status and vicinity to a dedicated pediatric center. When a head CT scan is performed the craniocervical junction should be included in the scan.

### Key action statement 7

In children with ventricular shunt who sustain a minor head trauma and have no PECARN predictors of traumatic brain injury (Table 2) and no other risk factors from history, clinicians should favor initial observation over routine immediate CT scan.

#### Action statement profile: KAS 7

**Table:** 

Aggregate evidence quality	B
Benefits	Limitation of exposure to risks related to radiation and possible need for sedation, as well as reduction in costs, for children at negligible risk of ciTBI who are already exposed to higher radiation doses due to underlying pathology
Risk, harm, cost	Negligible risk of missing a ciTBI Costs of observation over CT scan
Benefit-harm assessment	Benefits outweigh harms
Values judgments	Concern for unnecessary radiation and potentially high accumulated radiation doses in children already exposed to repeated CTs for their underlying condition
Intentional vagueness	None
Role of patient preference	None
Exclusion	Patients with GCS < 15 or signs and symptoms of traumatic brain injury
Strength	Moderate recommendation
Difference of opinion	None

### Accompanying text

The purpose of this statement is to offer guidance on decision-making about whether to order a head CT scan in children with ventricular shunts who present to the ED following a minor blunt head trauma and have no signs or symptoms of traumatic brain injury. The PECARN rule does not apply to this group of patients. These children along with those with known brain tumors, preexisting neurologic disorders, bleeding disorders, or neuroimaging performed at a transferring hospital were excluded from the PECARN rule study [3].

The presence of a ventricular shunt may potentially increase the risk of intracranial hemorrhage following head trauma by stretching the bridging veins or cortical arteries that normally adhere to the inner surface of the dura [105–108]. This potential risk has led to the common practice of ordering a cranial CT scan for most children with VP shunt presenting to the ED following a minor head trauma [109]. However, it must be taken into account that children with ventricular shunt are exposed to repeated CT scans for their underlying condition and additional CT scans following a head trauma contribute to the cumulative risk of repeated radiation exposures [110].

A recent a priori-planned secondary analysis [109] of the PECARN dataset [3] is the only prospective study that provides a risk estimate of ciTBI in children with ventricular shunt presenting to the ED following a minor head trauma. The study included 98 patients with ventricular shunt and 39,634 patients without shunt who presented to the ED with a GCS ≥ 14 within 24 h following a blunt head trauma. Patients with and without ventricular shunt were comparable for baseline clinical characteristics. Of the patients with ventricular shunt 14% had signs of altered mental status, 19% had a non-frontal hematoma, while a history of vomiting, loss of consciousness and severe mechanism of injury was present in 16%, 10% and 9% of patients respectively. The prevalence of ciTBI in patients with ventricular shunt was similar to patients without shunt, 1% (1 out of 98 patients) and 0.9% (346 out of 39,619 patients) respectively, with a difference of 0.1% and 95% CI of −0.3-5%. The one child with a ventricular shunt who had a ciTBI was a 10-year-old boy who walked into a stationary object and had no PECARN traumatic brain injury predictors. However, this patient had a known chronic subdural hematoma that was larger after the head trauma compared with previous CT, leading to neurosurgical hematoma evacuation. Even though the small number of patients with ventricular shunt in the study limits the ability to make precise risk estimates, the CIs around the differences between groups were relatively narrow, even after use of accepted statistical methods for rare outcomes (low prevalence rates). While 46% of patients with ventricular shunt underwent a cranial CT the remaining 54% received standardized clinical follow up in order to meet the ciTBI patient centered outcome definition. Of the 43,498 patients enrolled in the parent study [3] 2912 (7%) were excluded for missing information about the presence or absence of ventricular shunts. However selection bias was very unlikely to affect the results of the analysis given the very low prevalence of children with ventricular shunt in the overall enrolled population (0.2%).

Despite the small number of patients, this is to date the largest available cohort and the first study providing a risk estimate of ciTBI in children with ventricular shunt following minor head trauma. Due to the similar risk of ciTBI in children with and without ventricular shunt, clinicians should not base neuroimaging decisions purely on the presence of the shunt. In these children routine immediate cranial CT may not be indicated in the absence of other risk factors for TBI. In addition, the risk of a delayed diagnosis of a ciTBI is further reduced by close observation in the ED [111, 112].

### Key action statement 8

Clinicians should avoid routine repeated CT scan in children with GCS 14–15 and a non-clinically significant intracranial injury on initial CT. Decision on repeated CT should be based on a careful monitoring of the neurological status and consultation with the neurosurgeon.

#### Action statement profile: KAS 8

**Table:** 

Aggregate evidence quality	C
Benefits	Limitation of exposure to risks related to radiation and possible need for sedation, as well as reduction in costs
Risk, harm, cost	Potential delayed identification of injury progression Costs of observation over CT scan
Benefit-harm assessment	Benefits outweigh harms
Values judgments	None
Intentional vagueness	Clinically significant intracranial injury based on clinical judgment
Role of patient preference	None
Exclusion	Patients with clinically relevant intracranial injury on initial CT
Strength	Weak recommendation
Difference of opinion	None

### Accompanying text

The purpose of this statement is to offer guidance on decision to repeat CT in children with positive initial CT for a non-clinically significant intracranial injury.

The literature provides limited data to define the optimal management of children with a non**-**clinically significant intracranial injury not requiring neurosurgery after initial assessment [113–117].

Most of these children undergo repeat CT 24–48 h following initial CT to monitor the injury evolution in order to identify a possible deterioration on imaging before clinical deterioration occurs, in order to anticipate the need of medical or surgical treatment and improve patient outcome [114, 116, 117]. In children with minor head trauma the rate of injury progression on repeat CT varies between 6.6% and 26% [113, 114, 117–120]. However, it is unclear how often the progression on imaging determines changes in the medical or surgical management, [113, 119] or whether a timely surgical intervention based on imaging progression, before clinical deterioration occurs, leads to improved patient outcome and reduction in healthcare costs [114].

Available data show that a change in management follows repeated CT only in a small minority (0–2%) of children with a non-clinically significant injury on initial imaging. This small percentage refers to children who underwent repeat CT based on signs/symptoms of neurologic deterioration [113, 115, 118–121].

The pediatric studies on this topic widely differ for the characteristics of the population included, the severity of the head injury and the final outcome [113–122]. Children with moderate or severe head injury are more likely to undergo a change in management following results of repeated CT scan [119]. Some authors recommend a repeated CT in the presence of 3 or more intracranial lesions, a mass effect, an intraventricular hemorrhage, an epidural hematoma on initial CT [116]. An epidural or subdural hematoma, the presence of cerebral edema, intraparenchymal hemorrhage seem to be associated with a higher risk of evolving and a higher likelihood to lead to a change in management, including surgery [114].

According to several reports stable or clinically improving patients with a GCS of 14–15 and non-surgical intracranial lesions on initial imaging do not need to undergo routine repeated CT, as its results are highly unlikely to lead to a change in management [113, 118, 119, 121–123].

A retrospective study assessing the usefulness of routine repeated CT scan in 136 children 2–18 years of age with non-clinically significant intracranial lesion on initial CT and a GCS of 13–15 found an improvement on repeat imaging in 78%, stable injuries in 11% and a progression in 11%. None of these patients required neurosurgery. Conversely, all the three patients who underwent repeated CT because of clinical deterioration showed injury progression and two of them needed neurosurgery [119]. Another study of patients 0–18 years of age, including a subgroup with a GCS of 13–15, reported no need of management variation in all the patients with minor head trauma who underwent routine repeated CT scans [122]. A study of 120 patients aged 1 week to 17 years with GCS 14–15 following head trauma found a progression of injury on routine repeated CT in 7 (6.6%) patients, with two patients requiring neurosurgery for an epidural hematoma. While one of these patients presented nausea and vomiting the other was reported to be asymptomatic. None of the patients who did not undergo routine repeated CT scans presented neurologic deterioration or signs and symptoms of potential injury progression [118]. A retrospective study including 257 children younger than 15 years with minor head trauma and an intracranial injury on initial CT reported that three patients (1%) underwent neurosurgery after repeated CT showing a progression of the injury. However, all three patients presented a deterioration in their GCS before receiving a repeat CT [113]. Another study found that only one patient, out of 47 with intracranial injury following head trauma, needed urgent neurosurgery following repeated CT, performed because of developing signs of increased intracranial pressure [121]. In children with initial CT showing a non-surgical intracranial injury, close clinical monitoring including repeat assessment of neurologic status seems to be the best approach to decide when a repeated CT is necessary [118–121].

### Key action statement 9

In children presenting to the ED with minor head trauma clinicians should not use skull radiographs as a screening tool for clinically important traumatic brain injuries.

#### Action statement profile: KAS 9

**Table:** 

Aggregate evidence quality	B
Benefits	Avoidance of costs, additional radiation and reduction of time spent away from the department for a test that is poorly accurate in identifying intracranial injuries and bears the risk for misdiagnosis
Risk, harm, cost	Potential risk for rise in CT rate
Benefit-harm assessment	Benefits outweigh harms
Values judgments	None
Intentional vagueness	None
Role of patient preference	None
Exclusion	None
Strength	Strong recommendation
Difference of opinion	None

### Accompanying text

The purpose of this statement is to offer guidance on decision-making about the use of skull X-rays as a screening tool for the diagnosis of intracranial injury in children following a minor head trauma.

Historically, in the absence of readily available CT scanners and high-quality clinical decision rules, skull X-ray was used to categorize patients with minor head trauma into high and low risk for traumatic brain injury, based on the detection of a skull fracture.

Prospective observational studies and meta-analysis have demonstrated the association of a skull fracture, identified on a skull X-ray, with a significantly higher risk of intracranial injury in both pediatric and adult patients [124–127]. Skull radiographs have the advantages to expose children to less radiations compared with CT and to require no sedation.

However, the same studies [124–127] have shown that a significant number (up to 50%) of intracranial injuries can occur in the absence of a skull fracture. In addition there is a high likelihood that both junior doctors and qualified emergency physicians may misread skull radiographs when compared with a radiologist [125, 128, 129].

For these reasons skull radiographs have generally been not recommended as a screening tool for intracranial injury when CT is readily available [94, 124, 125]. A retrospective study in the UK monitoring practice following the abolition of skull x-rays on the management of pediatric head injuries in children older than one year of age showed that skull radiographs could safely be abandoned in these patients. They found a slight increase in the CT scan rate (from 1% to 2.1%) following abolition of skull x-rays, with no change in the positive CT pick up rate, no significant change in admission rate and a slight decrease in the radiation dose per head trauma [130].

Skull X-rays, however, have been recommended by many authors and guidelines in the assessment of young infants with a large isolated scalp hematoma as the sole manifestation of head trauma [124, 131–135]. These otherwise asymptomatic infants have shown to be at greater risk for skull fracture, and therefore for intracranial injury [124, 131, 135, 136]. Negative skull radiographs in these patients were proposed to obviate the need to perform a CT scan as the risk for TBI is reduced, although not absent.

In the era of high quality clinical prediction rules for pediatric head trauma, and patient centered outcomes, i.e. TBI that require acute intervention such as neurosurgery, intensive care or prolonged hospitalization, rather than the sole presence of an intracranial injury on CT, [3, 92, 93] better estimates of the risk of ciTBI are available for asymptomatic infants with isolated scalp hematoma.

A recent a priori-planned secondary analysis [137] of the PECARN head injury rule study [3] is the largest prospective study including 2998 children younger than two years with isolated scalp hematoma. A ciTBI occurred in 12 patients (0.4%; 95% CI 0.2% to 0.7%) and none required neurosurgery (95% CI 0% to 0.1%). Of 570 patients (19.0%) for whom CTs were obtained, 50 (8.8%; 95% CI 6.6% to 11.4%) had a TBI on CT. Younger age, non-frontal scalp hematoma location, increased scalp hematoma size, and severe injury mechanism were independently associated with traumatic brain injury on CT. These clinical factors, rather than a skull XR should guide the choice on CT scan performance, considering that of children who are imaged with CT and have skull fractures, approximately 30% have intracranial injuries [unpublished PECARN data, courtesy of Prof Nathan Kuppermann].

While skull X-rays could be a good diagnostic tool, in expert hands, for the identification and definition of fractures in children with large overlying hematomas, the advent of point-of-care ultrasound is likely to replace skull radiographs for this purpose, as characteristics of the fracture can be better studied. Point-of-care ultrasound has the advantages of avoding the patient both exposure to radiation, and time spent away from clinical observation and monitoring in the ED.

However, skull X-rays maintain a role as part of the skeletal survey in children presenting with suspected non-accidental injury [94, 138].

### Key action statement 10a

Clinicians should not routinely use trans-fontanelle ultrasound for diagnosing intracranial injuries in infants presenting to the emergency department following a trauma to the head.

#### Action statement profile: KAS 10a

**Table:** 

Aggregate evidence quality	D
Benefits	Avoiding to potentially miss an intracranial injury due to poor test accuracy
Risk, harm, cost	Potential risk for rise in CT rate
Benefit-harm assessment	Benefits outweigh harms
Values judgments	None
Intentional vagueness	None
Role of patient preference	None
Exclusion	None
Strength	Weak recommendation
Difference of opinion	None

### Accompanying text

The purpose of this statement is to offer guidance on decision-making about the use of trans-fontanelle cerebral ultrasound as a screening tool for the diagnosis of intracranial injury in infants following a minor head trauma.

Trans-fontanelle cerebral ultrasound is a bed-side, easy-to-use, and cheap radiation free test that does not require sedation to be properly performed. Although it is an accurate test to identify neonatal and perinatal brain injuries, the very limited ability to assess peripheral sub-cranial regions makes trans-fontanelle ultrasound inaccurate to identify extra-axial hematomas in infants with head trauma.

We could find only one prospective study where trans-fontanelle ultrasound was used as first neuroimaging test in 118 infants younger than 12 months who had a skull fracture on X-rays and an adequate size fontanelle. Of these, 2 patients were diagnosed with intracranial alterations and received a head CT scan that confirmed a small epidural hematoma in both cases, which did not need neurosurgery. No complications were found at the follow up visit at 2 months post injury in the remaining 116 patients and none required readmission [139].

Despite the promising results of this study the GDG agreed that these data were not sufficient to support the use of trans-fontanelle ultrasound in infants with head trauma in the era of PECARN clinical prediction rules.

### Key action statement 10b

Clinicians may choose to use point-of-care ultrasound for the identification of skull fractures and the definition of their characteristics (e.g. depression, diastasis) in children with minor head trauma.

#### Action statement profile: KAS 10b

**Table:** 

Aggregate evidence quality	B
Benefits	Avoiding radiation exposure and need for sedation
Risk, harm, cost	Misdiagnosis of skull fracture
Benefit-harm assessment	Benefits outweigh harms
Values judgments	None
Intentional vagueness	None
Role of patient preference	None
Exclusion	Patients with GCS < 14
Strength	Moderate recommendation
Difference of opinion	None

### Accompanying text

The purpose of these statements is to offer guidance on decision-making about the use of ultrasound in the diagnosis of skull fractures in children following a minor head trauma.

As reported above, under the recommendation on the use of skull x-rays (Key Action Statement 9), skull fractures are inaccurate predictors of the presence of traumatic brain injuries and skull ultrasound should not be used as a screening tool for intracranial injuries, but rather to identify a skull fracture and define its characteristics.

Point-of-care ultrasound is increasingly being used in the emergency setting to provide quick bedside information in the assessment of various fractures [140]. Ultrasound is a safe, quick and non invasive test that does not involve exposure to ionizing radiation and can be performed in the ED, allowing observation and monitoring to continue in a safe environment.

Although current management of pediatric minor head trauma is based on the use of accurate clinical prediction rules to guide the choice on CT or observation, the use of ultrasound may be helpful for the following reasons:

- to favor a more rapid CT decision making in children with a scalp hematoma if a depressed and/or diastatic skull fracture, which is likely to need neurosurgery independently of the risk of traumatic brain injury, is identified on ultrasound examination
- to plan a better follow up for children found to have a skull fracture with respect to the rare, but significant late complication of a “growing skull fracture”, usually occurring during infancy and early childhood [141, 142]. The tear of the dura that might be associated with a skull fracture may lead to the herniation of brain tissue or arachnoid membrane through the fracture margins with the growth of the skull, resulting in a leptomeningeal cyst or “growing skull fracture”. This condition needs to undergo surgical repair that includes resection of the leptomeningeal cyst and degenerated brain tissue, repair of the dural defect, and cranioplasty [141, 142].
- to tailor the advice given on discharge with respect to sport and play in children found to have a skull fracture on ultrasound, who may not require a CT scan based on clinical prediction rules

Various studies investigated the accuracy of skull ultrasound in identifying skull fractures in children following a minor head trauma compared with CT findings (gold standard) [143–147]. These studies showed varying sensitivity (ranging from 82% to 100%), with wide confidence intervals. Specificity ranged from 94% to 100%. Overall, diagnostic accuracy based on total pooled data from four studies [143–145, 147] including a total of 185 patients with 50 skull fractures found a sensitivity of 94% (95% CI 84–98%), a specificity of 96% (95% CI 92–98%), a positive likelihood ratio of 25 (11–60) and a negative likelihood ratio of 0.1 (0.0–0.2) [143]. However these studies showed variability with respect to the characteristics of the included population, the level of training of physicians performing the ultrasound, the technique used and the blinding with the results of the CT. Only one study assessed the agreement between physicians with a different level of expertise on ultrasound, finding a good agreement rate [143]. However, no study has assessed the usefulness of skull ultrasound in children younger than 2 years of age with a non-frontal scalp hematoma, which is an intermediate PECARN risk factor for clinically important traumatic brain injury [3]. In summary, point-of-care ultrasound can be used to detect skull fractures in children with minor head trauma by trained providers, however the evidence from available studies is insufficient to recommend its routine use in clinical practice, where the use of selective CT based on accurate clinical prediction rules remains the gold standard [3, 103].

### Key action statement 11

Clinicians should not routinely use near-infrared spectroscopy (NIRS) technology devices to screen for intracranial hematomas in the assessment of children presenting to the emergency department following a trauma to the head.

#### Action statement profile: KAS 11

**Table:** 

Aggregate evidence quality	C
Benefits	Avoiding possible misdiagnosis of intracranial injuries (although the test is radiation free and can be performed without sedation)
Risk, harm, cost	Missing possible opportunities for anticipating CT scan decision-making in some patients
Benefit-harm assessment	Benefit/risk or cost ratio unclear based on available evidence.
Values judgments	Further research is needed to clarify the possible usefulness of near infrared devices in conjunction with the PECARN algorithms for optimizing patient selection for CT scan.
Intentional vagueness	None
Role of patient preference	Not applicable
Exclusion	Children with head trauma >12 h, large scalp hematomas or lacerations, thick hair (limitation to the use of NIRS devices)
Strength	Weak recommendation
Difference of opinion	None

### Accompanying text

The purpose of this statement is to offer guidance on the use of near infrared technology in children presenting to the ED with blunt head trauma as an aid to clinical assessment in the decision-making on whether to order a CT scan.

The use of near infrared spectroscopy (NIRS) technology has shown good accuracy for the detection of traumatic intracranial haematomas in adults [148–154], with a sensitivity ranging from approximately 70% to 100% and specificity equal or greater than 80%.

NIRS technology identifies intracranial haematomas by comparing the optical density of infrared light absorption between symmetrical regions in the two sides of the head. The extravascular blood of the haematomas absorbs NIR light more than intravascular blood. This is due to the higher concentration of heme-based proteins, such as hemoglobin in the hematoma compared with normal brain tissue, where blood is contained within vessels. Under normal circumstances brain’s absorption is symmetrical. When a haematoma is present on one side of the brain a difference in light absorption is detected and recorded by the NIRS device. The examination includes a set of four pairs of measurements of four regions of the brain (frontal, temporal, parietal, and occipital), where the device is placed in sequence on the left and right side over pre-selected locations.

Two studies included a mixed adult and children population [155, 156]. However, neither the exact number of children included, nor separate pediatric results were reported.

Only three studies have assessed the feasibility and/or diagnostic accuracy of portable hand-held non-invasive NIRS devices specifically in children with head injury [157–159]. Interpretation and applicability of their results is affected by significant limitations.

The study by Coksun et al. [157] included 161 children who underwent a head CT for head trauma at the Emergency Service of Ankara Training and Research Hospital, in Turkey. All the patients underwent both the study test (NIRS) and the gold standard (CT scan). The assessors of the index test and reference standard were blind to the results of the other test. They reported a sensitivity of 86% (CI 95% 60–96), a specificity of 64% (CI 95% 56–71), a positive predictive value of 18.5% (CI 95% 11–29.6) and a negative predictive value of 97.9% (CI 95% 92.7–99.4%). However, patients’ characteristics and feasibility of the NIRS technique assessment (successful completion rate and completion time) were not reported.

A small study including 28 children admitted to a Paediatric Intensive Care Unit, who received a CT scan as part of their routine clinical care (for both traumatic and non-traumatic conditions), showed a NIRS completion rate of 79%, and a completion time up to 15 min [158]*.* The sensitivity and specificity of NIRS to detect intracranial haemorrhages were 100% and 80%, respectively. The positive and negative predictive values were 80% and 100%, respectively. The operator of the NIRS device was not blind to whether the CT was normal or abnormal. The small sample size, the difference in population compared with children presenting to the ED with head trauma and the lack of blinding in performing the NIRS assessment are important limitations to this study.

A pilot study conducted in a pediatric ED in Italy [159] included 110 children with minor head injury at moderate or high risk of ciTBI, as per PECARN algorithms [3]. The study showed a NIRS test completion rate of 94% and a time to completion of 4.4 ± 2.9 min, with a slightly longer time for children younger than two years, 5.5 ± 3.1 min (due to their lack of cooperation and need of repeat measurements). A CT scan was performed in only 18 (17.5%) children. The NIRS operator was blind to whether a CT was going to be performed and the radiologist who read the CT scans was unaware of the NIRS test results. The main limitation of this study is the small sample size and the low number of CT (reference test) performed. The insufficient number of positive CT scans is responsible for the very wide confidence intervals of diagnostic accuracy measures. The sensitivity, specificity, positive and negative predictive values for identification of intracranial haematomas on CT scan were 100% (95% CI, 20.7–100), 100% (95% CI, 81.6–100), 100% (95% CI, 20.7–100) and 100% (95% CI, 81.6–100) respectively. The sensitivity, specificity, positive and negative predictive values for identification of ciTBI (determined by either CT or telephone follow up for children who did not receive a CT) were 100% (95% CI, 20.7–100), 93.1% (95% CI, 86.5–96.6), 12.5% (95% CI, 2.2–47.1) and 100% (95% CI, 96.1–100) respectively.

Intrinsic limitations of NIRS technology to identify traumatic intracranial haemorrhages should also be noted. First of all the detection limits of the device for intracranial hematomas are a volume of blood ≥3.5 mL, within a depth of 2.5 cm of the brain surface. This affect accurate identification of deep hematomas or contusions, or very small superficial bleedings. Second, bilateral hematomas cannot be reliably identified by near-infrared technology, as the technique relies on comparison of light absorption between the two hemispheres. Third, the utility of NIRS in detecting subacute or chronic haematomas is limited to the first 12 h following the injury, since this technology is based on the absorption characteristics of acute bleeding and haemoglobin breakdown products that develop in the following hours do not have the same absorption characteristics. Fourth, scalp haematomas are confounding factors for near-infrared technology measurements. Blood contained within a scalp hematoma can alter the difference in optical density and cause a false-positive result. Despite symmetrical measurements can be performed at the edges of the haematoma, this limit questions the applicability of near-infrared technology to the challenging group of children younger than 2 years of age with large isolated scalp haematomas. Furthermore, thick hair may affect examination performance, while cervical collars may limit the ability to carry out the measurements in the occipital pair of locations.

NIRS technology is not meant to be used in isolation and may be helpful in selected group of patients. Further research is needed to clarify the possible usefulness of this technology in conjunction with the PECARN algorithms with the purpose of optimizing CT scan use.

### Key action statement 12a

ED physicians should favor initial observation over CT scan for children at intermediate-risk of clinically important traumatic brain injury (ciTBI) according to the age-appropriate PECARN algorithms, especially in the presence of isolated findings.

#### Action statement profile, KAS 12a

**Table:** 

Aggregate evidence quality	B
Benefits	Limitation of risks related to radiation and possible need for sedation, and reduction in costs, for children at negligible risk of ciTBI
Risk, harm, cost	Negligible risk of missing a ciTBI Costs of observation over CT scan
Benefit-harm assessment	Benefits outweigh harms
Values judgments	EDs adopting this strategy should have internal guidelines/protocols in place for: - close monitoring of head injured children during observation - provision to families of detailed discharge instructions on when to return to the ED
Intentional vagueness	None
Role of patient preference	Parents preference should be considered
Exclusion	Children with bleeding disorders, underlying neurologic risk factors and suspect child abuse
Strength	Strong recommendation
Difference of opinion	None

### Accompanying text

The purpose of this statement is to offer guidance on decision-making on observation as an alternative to CT scan in children presenting to the ED with minor head trauma. In these patients clinical observation prior to CT decision-making is recommended as an effective approach by the American Academy of Pediatrics “Choosing Wisely” Campaign [160]. This clinical management strategy has the potential to decrease unnecessary CT scans while minimizing the risk of missing a ciTBI. Observation allows time for the child’s symptoms and/or signs to improve or evolve, leading to a selected CT use in those patients with lack of improvement or worsening of symptoms and signs during observation.

The PECARN algorithms recommend observation as an alternative to CT scan for children at intermediate risk of ciTBI, taking into consideration other factors such as physician experience, the presence of multiple versus isolated findings, worsening symptoms and signs during observation, parental preference and age < 3 months [3].

A secondary analysis of the PECARN head injury parent study showed that clinical observation before CT decision making resulted in a safe and potentially effective strategy to manage a subset of children with minor head trauma [111]. The study including over 40,000 children found that in the 14% who were observed, the CT scan rate resulted significantly lower (11% relative reduction from the baseline 35% CT use rate) compared with children who were not observed before CT decision making. There was no increase in the rate of significant traumatic brain injuries. The odds of obtaining a CT remained significantly lower in children who were observed even after adjusting for age, factors associated with TBI (i.e. mechanism of injury, symptoms and signs at presentation) and hospital center (adjusted odds ratio 0.53 [95% CI: 0.43–0.66]). As expected, the rate of CT use was lower for patients whose symptoms improved during the period of observation.

A more recent single-center study on 1381 prospectively enrolled children with minor head trauma presenting to a tertiary care ED found that ED observation time was associated with a time-dependent reduction in cranial CT rate in all three PECARN risk groups, with no delay in the diagnosis of ciTBI [112]. Overall 676 (49%) patients were observed in the ED (49% very low risk, 45% intermediate risk and 6% high risk) and 272 (20%) had a CT performed. Children whom clinicians chose to observe presented to the ED sooner after their head injury than those who were not observed. The CT rate was significantly lower for children who were observed (5% observed versus 34% non observed). All 8 children with a ciTBI had an immediate CT.

Due to the relatively small number of patients with ciTBI, both studies had limited ability to identify possible uncommon negative outcomes of observation, i.e. patients who might experience a clinically relevant delay in the diagnosis of ciTBI.

A previous multicenter RCT showed no significant difference in mortality and neurologic disability at three months after injury between patients with minor head trauma who underwent immediate CT versus in-hospital observation. Both group of patients were similarly satisfied with the care received [161]. This study, however, included only patients older than 6 years, (920 of the 2602 enrolled patients (35%) were <15 years of age) and had different inclusion criteria (i.e. ED presentation within 24 h since injury, confirmed or suspected loss of consciousness and/or amnesia, a GCS of 15, a normal neurologic examination and no associated injuries that required admission). A prospective cost effectiveness analysis performed on the data of the same RCT showed that the immediate CT strategy was less expensive than admission for observation [162]. However, the radiation-induced risk of cancer was not taken into account. In addition the exclusion of children younger than 6 years significantly limits the applicability of the study findings to the pediatric age.

While further studies are needed to evaluate the tradeoff in health care costs between observation-associated longer ED stays and a reduced CT rate, observation appears to be the most effective strategy for children at PECARN intermediate risk, for whom the need for cranial CT may not be obvious at the time of initial evaluation. To further support clinicians decision-making for children at PECARN intermediate-risk several sub-analysis of the parent study have reported on the risk of ciTBI associated with the presence of isolated intermediate-risk findings (see Table 2) [137, 163–167].

For this group of patients parental preference should also be taken into account in the clinical decision-making process. The results of a recent survey showed that parents seem to prefer observation in the ED over immediate CT in the management of their child’ s minor head trauma [168]. However, future work will need to clarify the best method of risk communication to patients and their families with respect to ED decisions on diagnostic imaging.

The decision to forgo CT scan after an ED observation period will depend on physician experience, risk tolerance and shared decision making with patients, and their families.

### Key action statement 12b

ED physicians who elect to observe previously-healthy children >3 months of age at PECARN intermediate risk of ciTBI following a minor head trauma, should observe these patients for a minimum of 4–6 h from the time of injury.

#### Action statement profile, KAS 12b

**Table:** 

Aggregate evidence quality	C
Benefits	Minimization of risk of missing a ciTBI while avoiding unnecessary radiation exposure Avoidance of possible need for sedation Saving CT scan related costs
Risk, harm, cost	Longer ED length of stay Costs associated with observation Reduced throughput for other patients
Benefit-harm assessment	Benefits outweigh harms
Values judgments	EDs adopting this strategy should have internal guidelines/protocols in place for: - close monitoring of head injured children during observation - provision to families of detailed discharge instructions on when to return to the ED
Intentional vagueness	Time interval of 4–6 h was chosen based on the scant available evidence, as no specific duration has shown to be safer
Role of patient preference	Should be considered
Exclusion	Moderate-severe head trauma
Strength	Weak
Difference of opinion	None

### Accompanying text

The purpose of this statement is to offer guidance on the duration of clinical observation for children with minor head trauma, whom ED physicians decide to observe prior to CT-scan decision making.

Although rarely, children with minor head trauma may present delayed clinical decompensation due to the evolution of their intracranial injury. While observation appears to be an effective strategy to optimize the selection of patients who need to undergo CT scan, its duration should ensure the early identification of patients who may present delayed deterioration.

Although two recent large prospective studies assessed the effectiveness of clinical observation as an alternative to immediate CT scan, neither of them could define an optimal observation period [111, 112]. While in one study the duration of observation was not reported [111], the other showed an average 70% reduction in CT rate for every hour of ED observation, after adjustment for other patient and provider factors [112]. In this study, the median time between the injury and CT decision making for observed patients was approximately 4 h. Although none of the 644 children who were observed without receiving a CT returned for a delayed ciTBI, the study was not designed to determine the optimal period of observation before CT decision-making. Due to the small number of ciTBI (8 patients) in the overall study population and the lack of a structured clinical follow-up, rare but possible cases of delayed ciTBI may have been missed.

A recent population-based study found that the incidence of delayed diagnosis of intracranial hemorrhage (i.e. not apparent until ≥6 h after injury) is rare [169]. This retrospective study included nearly 18,000 patients presenting with minor blunt head trauma to any ED in the Calgary Health Region, in Canada, over an 8-year study period. Minor head trauma was defined as absence of loss of consciousness greater than 1 min duration or amnesia, a GCS of 15 and normal neurologic examination; children were considered to have delayed diagnosis of intracranial hemorrhage if they were reported to be awake and alert with normal neurologic examination for at least 6 h after the injury and had any type of intracranial hemorrhage diagnosed at CT or MRI after this interval. Two children had a delayed diagnosis of intracranial hemorrhage associated with deterioration in level of consciousness at the time of diagnosis (0.0%, upper limit of 95% CI: 0.02%). Eight children had a delayed diagnosis of intracranial hemorrhage, which was not associated with deterioration in level of consciousness (0.03%, 95% CI: 0.01–0.07%). The calculated incidences of delayed diagnosis of intracranial hemorrhage with and without deterioration in level of consciousness were 0.14 and 0.57 cases per 100,000 children per year, respectively.

An Italian study focusing on the use of CT scan in children at PECARN intermediate risk of ciTBI reported one case of delayed intracranial injury diagnosis in 269 patients who were observed without receiving a CT scan [104]. In this study monitoring for delayed diagnosis was carried out by reviewing ED return visits within 2 weeks since initial presentation and by telephone contact (possible in 80% of cases) for patients who did not return to the ED. The child with delayed diagnosis of intracranial hemorrhage was a previously healthy 4-year-old boy who initially presented for repeated vomiting and mild headache 16 h after sustaining an occipital head trauma following a non-severe mechanism of injury. He was discharged after a 3 h observation during which he maintained a GCS of 15 and a normal neurologic examination with complete symptoms resolution. The CT scan performed the following day, when he represented for balance problems, revealed an extradural haematoma that was managed conservatively during his 2-night admission.

Although current evidence does not clarify what is the optimal period of observation, based on available data, the risk of delayed diagnosis of a ciTBI seems to be very low following observation up to 4 to 6 h after injury. The GDG agrees this is a reasonable duration to allow symptom progression or resolution in a monitored environment. While this duration minimizes, but does not eliminate the risk of potentially missing a ciTBI, the GDG highlights the importance of careful discharge instructions to reduce delay to diagnosis after discharge.

### Key action statement 12c

ED physicians who elect to observe infants younger than 3 months at PECARN intermediate risk of ciTBI following a minor head trauma should consider to observe them for 24 h.

#### Action statement profile, KAS 12c

**Table:** 

Aggregate evidence quality	D
Benefits	Minimization of risk of missing a ciTBI while avoiding unnecessary radiation exposure Avoidance of possible need for sedation Saving CT scan related costs
Risk, harm, cost	Longer ED length of stay Costs associated with observation Reduced throughput for other patients
Benefit-harm assessment	Benefits outweigh harms
Values judgments	EDs adopting this strategy should have internal guidelines/protocols in place for: - close monitoring of head injured children during observation - provision to families of detailed discharge instructions on when to return to the ED Clinical experience was used in making this judgment while recognizing that extensive data from studies are lacking
Intentional vagueness	None
Role of patient preference	Should be considered
Exclusion	Moderate-severe head trauma
Strength	Weak
Difference of opinion	None

### Accompanying text

The purpose of this statement is to offer guidance on the duration of clinical observation for infants younger than 3 months with minor head trauma, whom ED physicians decide to observe prior to CT-scan decision making.

Infants younger than 3 months of age are known to be at higher risk for TBI even following minor falls [133, 167]. The PECARN head injury algorithms list age younger than 3 months as an additional factor to be considered when making the decision between immediate CT versus initial observation in the intermediate-risk group, because of the higher risk of TBI in this age group [3].

However, as reported in the accompanying text to KAS 12b, current evidence does not clarify what should be the optimal observation period for children following minor head trauma prior to making a final decision on CT.

Taking into consideration the higher risk of intracranial injury in this age group and their challenging medical assessment due to their limited ability to express symptoms the GDG agreed that children younger than 3 months, should be observed for a 24 h period when decision is made to forgo immediate CT. Although longer ED stays have resource implications and may reduce throughput for other patients, the small number of patients younger than 3 months presenting for minor head trauma is unlikely to significantly impact on ED workflow.

### Key action statement 12d

Children who require observation in the ED following a head trauma should be appropriately monitored by clinical staff who are qualified to deliver care to children.

#### Action statement profile, KAS 12d

**Table:** 

Aggregate evidence quality	D
Benefits	Early recognition of possible clinical deterioration in children, and provision of adequate care to meet the unique needs of children
Risk, harm, cost	None
Benefit-harm assessment	Benefits outweigh harms
Values judgments	Concern for possible deterioration that may be unrecognized and not addressed appropriately and timely by clinical staff not trained in the care of children Clinical experience was used in making this judgment while recognizing that data from studies are lacking
Intentional vagueness	The definition of “appropriate monitoring” may vary according to the patient clinical status; however, a minimum acceptable monitoring during observation is reported in the accompanying text
Role of patient preference	None
Exclusion	None
Strength	Weak recommendation
Difference of opinion	None

### Accompanying text

The purpose of this statement is to offer guidance on the care that should be provided to children requiring observation in the ED following a head trauma. These patients may be observed in the ED for different reasons. Observation prior to CT scan decision making helps better select patients who need a CT scan, based on the evolution of their signs and symptoms. Observation after a CT may obviate inpatient admission in children with persistent symptoms despite a negative CT scan or in patients with small intracranial injuries amenable of conservative treatment according to neurosurgical advice [111, 112, 161, 170, 171].

Retrospective studies conducted in tertiary care pediatric centers showed that children with closed head injuries who are observed in a dedicated observation unit following identification of very small intracranial hemorrhages and/or skull fractures on CT, or concussive symptoms are successfully discharged within 24 h in >95% of cases and rarely require readmission in the 72 h following discharge [170, 171].

All but one studies retrieved by our search strategy on the assessment of clinical observation were conducted in pediatric EDs [111, 112, 170, 171]. The only study that enrolled a mixed pediatric and adult population with minor head trauma presenting to 39 of 75 EDs in Sweden, did not specify whether pediatric patients were observed in dedicated pediatric facilities or in a different setting, and included only children >6 years of age [161].

Although it is well recognized that the care of pediatric patients, especially younger children, is better provided by healthcare professionals specifically trained in pediatrics, the level of training and availability of trained staff may vary in small centers. The care and monitoring of head injured children by personnel not adequately trained in pediatrics bears the risk for potential delayed recognition of clinical deterioration and is an indication for patient transfer to appropriate pediatric facilities.

Policies, regulations and training requirements to ensure that children who need observation following a head trauma are properly taken care of by clinical staff adequately trained in pediatrics are beyond the scope of these guidelines. Each institution should have local guidelines and protocols in place to provide this service or to transfer patients to the nearest referral facility within the local health system network.

Although the frequency of reassessments during observation may vary based on clinical judgment, the GDG advises for the following minimum requirements in children with GCS of 15:

- hourly assessment and documentation of GCS (or its pediatric version for pre-verbal children), pupil size and reactivity, as well as any change in post traumatic signs and symptoms, for the first 4 to 6 h of observation
- full set of vital signs at admission and discharge
- pain score (recorded using age-appropriate scales) at admission, after the necessary treatment and whenever lack of improvement or worsening of pain is reported.

The GDG agrees that cardiorespiratory monitoring should be considered for patients with altered level of consciousness (GCS <15), abnormal vital signs on initial assessment or while sleeping.

Neurological observations should be recorded more frequently in these patients. While evidence base data on overnight neurologic monitoring are lacking the GDG advises for waking the child up intermittently to assess the neurological status. Neurologic assessment should be performed hourly in the first 4–6 h and should then be individualized based on the time since injury, signs and symptoms on initial assessment and any deterioration occurring during observation.

The use of a dedicated electronic or paper-based observation chart is suggested for proper documentation.

### Key action statement 13

In children presenting to the ED following a minor head trauma and with a personal history of neurosurgical intervention other than isolated placement of a ventricular shunt, clinicians may require a neurosurgical consult, considering the type and time of the intervention, to help support CT-scan decision making.

#### Action statement profile: KAS 13a

**Table:** 

Aggregate evidence quality	D
Benefits	Optimizatin of patient selection for CT scanning Avoidance of risks related to unnecessary radiation exposure, possible need for sedation, and reduction in costs, for children who may have already been exposed to higher radiation doses due to underlying pathology
Risk, harm, cost	Cost of hospital neurosurgical resources
Benefit-harm assessment	Benefits outweigh harms
Values judgments	Clinical experience was used in making this judgment while recognizing that data from studies are lacking
Intentional vagueness	None
Role of patient preference	None
Exclusion	Previously healthy patients Children with moderate or severe head trauma
Strength	Weak recommendation
Difference of opinion	None

### Accompanying text

The purpose of this statement is to offer guidance on the request of neurosurgical consultation to support CT scan decision-making in children who present to the ED following a minor blunt head trauma and have a personal history of neurosurgical intervention, other than isolated placement of a ventricular shunt.

A history of prior cranial neurosurgical interventions may be intuitively associated with a higher risk of intracranial complications following head trauma. This risk may vary according to the underlying patient condition, the type and time of intervention. It is generally estimated to be higher in the first weeks or months following a craniotomy.

The PECARN rule does not apply to this group of patients, as children with known brain tumors and preexisting neurologic disorders were excluded from the PECARN rule study [3].

Our search strategy could not identify any relevant paper on the risk of TBI on CT or ciTBI in children with a history of previous neurosurgical intervention, even when no publication date limits were used. The present recommendation is therefore based on expert opinion within both the GDG and the Italian Society of Pediatric Neurosurgery. The contribution of the patient underlying condition, the type and time of neurosurgical intervention to the risk of intracranial injury may vary widely between patients, making it difficult to define a subgroup of children who can safely avoid CT scan following a minor head trauma. Considering the broad spectrum of risk variability for intracranial injury in these patients, as well as the different levels of experience and expertise that physicians working in ED may have in caring for these children, the GDG deemed it appropriate to advise for neurosurgical consultation based on clinical judgment, taking into account the clinical factors reported above.

### Key action statement 13b

ED physicians must discuss with a neurosurgeon the care of all children with traumatic injuries on CT scan (excluding uncomplicated isolated linear skull fractures).

For children presenting with severe head trauma ED physicians should alert a neurosurgeon as soon as possible, ideally prior to CT scan performance.

#### Action statement profile: KAS 13b

**Table:** 

Aggregate evidence quality	X
Benefits	Timely intervention of injuries requiring neurosurgery; appropriate monitoring and follow up plan for those amenable of conservative treatment
Risk, harm, cost	Use of hospital neurosurgical resources
Benefit-harm assessment	Benefits outweigh harms
Values judgments	When making this recommendation the GDG considered the increased availability in Italy of real-time digital imaging system for telemedicine neurosurgical consultation
Intentional vagueness	None
Role of patient preference	None
Exclusion	Patients with isolated uncomplicated linear skull fractures
Strength	Strong recommendation
Difference of opinion	None

### Accompanying text

The purpose of these statements is to offer guidance on neurosurgical consultation for the management of intracranial injuries in children presenting to the ED following a head trauma.

Recent evidence has definitely shown that patients with isolated, uncomplicated linear skull fractures are at extremely low risk of deterioration and need of neurosurgery and may be safely discharged form the ED, thus obviating the need for neurosurgical consultation [172, 173].

For children with intracranial injuries on CT scan an urgent neurosurgery consultation is necessary to ensure timely operative management, where appropriate, or to provide advice on the most appropriate monitoring and follow up for the patient. The intuitive beneficial practice of requesting a neurosurgical consultation in children with traumatic intracranial injuries on CT scan, translated into recommendation in previous pediatric guidelines and has become a standard of care, despite the lack of supporting scientific evidence [174, 175]. However, the most recent update of the NICE guidelines recommend to discuss with a neurosurgeon the care of all patients with new, surgically significant abnormalities on imaging, specifying that the definition of ‘surgically significant’ should be developed by local neurosurgical centers and agreed with referring hospitals, along with referral procedures [94]. This recommendation from the NICE guidelines, which provide guidance for both adult and pediatric patients, likely reflects the careful assessment of appropriate neurosurgical resources use for patients most in need of intervention, in organizations with limited neurosurgical availability compared with patient volume. We could not find any studies evaluating the yield of using a proposed a priori definition of ‘surgically significant’ intracranial injury on the optimization of neurosurgical referral in children with head trauma.

The largest and most recent multicenter epidemiological study on over 40,000 children presenting to the ED for head trauma provides useful estimates on the need of neurosurgical intervention in children with traumatic intracranial injuries on CT scan [2]. In this study, approximately 7% of children who underwent a head CT scan, i.e. nearly 3% of the overall study population, had traumatic findings other than isolated linear skull fractures. Of these, 17% needed neurosurgical intervention, corresponding to 0.5% of the overall study population.

Based on these data and considering the increasing availability in Italy of real-time digital imaging system for telemedicine neurosurgical consultation, the GDG enforces the widely recommended practice of requesting a neurosurgical consultation for all head injured children with intracranial injuries on CT scan other than isolated linear skull fractures. This is justified by the high need for neurosurgery in the low number of children diagnosed with traumatic intracranial injuries on CT.

Similarly, when the estimated risk of needing a neurosurgical intervention is very high based on clinical presentation, as in children with severe head trauma, early involvement of the neurosurgeon, even before CT scan is performed, is likely to shorten the time to and optimize the set up for a life-saving neurosurgical intervention if needed. The importance of early neurosurgical involvement has been demonstrated by the better outcome of severely head injured patients following direct pre-hospital transport to a dedicated trauma center, which constitutes the foundation of modern trauma systems [176–179]. According to the largest pediatric head trauma study mentioned above, children with severe head trauma showed intracranial injuries on CT scan in approximately 20% of cases, and nearly one third of these required neurosurgery [2].

### Key action statement 14a

ED physicians working in centers with no CT availability should transfer all children presenting with head trauma and either a GCS < 14 or at PECARN high risk for ciTBI to referral pediatric centers with neurosurgical capability.

#### Action statement profile: KAS 14a

**Table:** 

Aggregate evidence quality	A
Benefits	Timely performance of CT scan to patients at high risk of intracranial injury
Risk, harm, cost	Cost of transfer and use of hospital resources
Benefit-harm assessment	Benefits outweigh harms
Values judgments	None
Intentional vagueness	None
Role of patient preference	None
Exclusion	Children at PECARN intermediate or low risk group
Strength	Strong recommendation
Difference of opinion	None

### Key action statement 14b

ED physicians working in centers with no CT availability should consider to transfer children at PECARN intermediate risk for ciTBI to referral pediatric centers, preferably with pediatric neurosurgical capability. Decision to transfer should take into consideration the availability of resources for appropriate clinical monitoring, the age of the child (transfer should be preferred in children <3 months) and physician experience.

#### Action statement profile: KAS 14b

**Table:** 

Aggregate evidence quality	D (based on consensus)
Benefits	Timely performance of CT scan/ provision of appropriate monitoring during observation
Risk, harm, cost	Cost of transfer. Use of hospital resources. Possible overtriage
Benefit-harm assessment	Benefits outweigh harms
Values judgments	None
Intentional vagueness	None
Role of patient preference	Parents preference should be considered
Exclusion	None
Strength	Weak recommendation
Difference of opinion	None

### Accompanying text

The purpose of these statements is to provide guidance on the transfer of children with head trauma who present to an emergency facility where CT scan is not available.

Even though pre-hospital triage systems aim to directly transport to centers with neurosurgical facilities most of the head injured children [1, 178, 180–182], some patients may self-present to other facilities. In addition, patients initially classified as low risk may deteriorate during assessment.

In emergency facilities where CT is not available, indications for inter-hospital transfer of children with head trauma strictly reflect indications for CT head performance. While a CT scan is recommended for all children with a GCS < 14, the PECARN algorithms should be used as a decision-making tool in children with minor head trauma (i.e. GCS 14 or 15) [3] (see KAS 6 and Fig. 2). According to the PECARN algorithms children who present a GCS of 14 or other signs of altered mental status, a palpable skull fracture (for children ≥2 years) or signs of basilar skull fracture (for children <2 years) are at high risk of ciTBI. For these children a CT scan is recommended, given the nearly 5% risk of ciTBI in this group of patients. In children at intermediate risk of ciTBI this risk is approximately 1% and observation is considered an appropriate alternative to CT scan depending on physician experience, multiple versus isolated findings, worsening symptoms or signs during observation, age < 3 months and parental preference. As PECARN algorithms were derived and validated based on data from tertiary care highly resourced pediatric EDs (provided with CT, a dedicated observation unit and staff trained in pediatric care), the GDG advises ED physicians who work in a facility without CT to use a lower threshold for transferring PECARN intermediate risk patients, especially when <3 months of age. Children at higher risk of intracranial injury should be transferred directly to a pediatric center with neurosurgical capability. The GDG acknowledges that contingent limitations on transport related resources or a rapid deterioration in the patient’s clinical status may influence decisions on the destination facility.

### Key action statement 15a

ED physicians working in centers with CT capability but without neurosurgery must follow local healthcare system network guidelines for decision-making on transfer of children with moderate-severe head trauma to referral centers.

Each regional system needs to have guidelines and protocols in place to ensure safe, timely and appropriate inter-hospital transfer of these children.

#### Action statement profile: KAS 15a

**Table:** 

Aggregate evidence quality	X
Benefits	Optimization of patient care based on the most appropriate use of local resources
Risk, harm, cost	None
Benefit-harm assessment	Benefits outweigh harms
Values judgments	The GDG acknowledges the wide variability of healthcare system networks throughout the country, which is multifactorial in origin
Intentional vagueness	None
Role of patient preference	None
Exclusion	Children with minor head trauma
Strength	Strong recommendation
Difference of opinion	None

### Accompanying text

The purpose of this statement is to provide guidance on the transfer of children with moderate-severe trauma who present to centers with CT availability, but without neurosurgery.

As previously noted in these guidelines, in Italy the organization of formal trauma management systems is heterogeneous and fragmented, especially for pediatric trauma patients [17]. However, province/regional healthcare system networks are in place to centralize care of the most severely injured patients to specialized facilities with the best available resources and expertise. Each healthcare system network presents differences in the availability and distribution of resources, the organization of the integrated pre-hospital/hospital care, as well as peculiar geographical features.

Although field pre-hospital triage aims to directly transport the most severely head injured children to centers with pediatric neurosurgical capability [182], unstable patients may be diverted to the nearest facility if stabilization cannot be achieved pre-hospital. In addition, some patients with moderate head trauma may self-present to less specialized hospitals, while some patients initially classified as low risk may deteriorate during assessment.

After stabilization, the decision on timing and modality of transfer to the referral center with neurosurgical capability should follow local healthcare system network guidelines. While rapid transfer of the most severely head injured patients to definitive care is the common goal in all systems, each healthcare system network may have different transfer thresholds (secondary triage criteria) for children with moderate head injury. Secondary triage criteria should be agreed with referral centers and should be based on the best trade-off between locally available clinical and technical resources, transport-related resources and distance from the closest pediatric neurosurgical facility. Early contact with the referral neurosurgeon is key to optimize the management of these patients.

While recognizing the need for more structured and standardized trauma systems for pediatric patients throughout the whole country, the GDG encourages each health system network to adopt and disseminate clear guidelines and protocols for inter-hospital transfer of head injured children to referral centers and stipulate clear interfacility transfer agreements. Appropriate transfer of injured children is essential as studies have shown that injured children treated at designated pediatric trauma centers have significantly better outcomes than those treated at adult trauma centers or non-trauma centers, with the highest benefit for the most severely injured [179, 183–185]. Organization of pediatric trauma care delivery into formal trauma systems not only improves survival of severely injured children but also favorably impacts on their functional long term outcome [186, 187]. A trauma system is responsible for the entire patient pathway from pre-hospital care, through ED resuscitation and specialist emergency surgical intervention, to reconstruction of injuries and rehabilitation [186]. The benefits of centralized care of pediatric patients to designated trauma centers likely result from the combined benefits of pediatric medical specialists and healthcare professionals accustomed to dealing with the special needs of children within a continuum of care. Referral or designated pediatric trauma centers function as pediatric hospital hubs within the trauma network and have the responsibility for coordinating the management of severely injured patients within a regional area.

Although pediatric trauma remains the leading cause of death and disability in children older than one year of age, the small numbers of severely injured children make it challenging to retain an appropriate skill set even in tertiary care centers. The refinement and use of pediatric guidelines within each healthcare system network, in addition to pediatric specific trauma training, will help healthcare professionals deliver the best care to pediatric severely injured patients in both referring and referral hospitals. The use of high-fidelity simulation can significantly improve clinicians’ skills and comfort in dealing with severely injured children [188].

### Key action statement 15b

In centers with CT availability, but without neurosurgery, ED physicians may perform a head CT scan of children with moderate-severe head trauma, after stabilization, only if it does not delay transfer to the definitive care referral center and provided that images are of good quality and can easily be transferred to the referral center.

#### Action statement profile: KAS 15b

**Table:** 

Aggregate evidence quality	D (based on consensus)
Benefits	Avoiding delays in transfer of severely head injured children to facilities able to provide definitive care. Limiting duplication of scans for low quality images.
Risk, harm, cost	None
Benefit-harm assessment	Benefits outweigh harms
Values judgments	The GDG took into account the increasing number of hospitals using teleradiology in making the reccomendation
Intentional vagueness	None
Role of patient preference	None
Exclusion	Children not severely injured
Strength	Weak recommendation
Difference of opinion	None

### Accompanying text

The purpose of this statement is to provide guidance on the timing of head CT imaging in children with moderate-severe head trauma who present to centers with CT availability, but without neurosurgical facility.

Early arrival of severely injured patients to an appropriate trauma center has been shown to be associated with improved outcomes [189]. Centers with no neurosurgical facility should make any effort to reduce delay to definitive care in head injured children who meet local transfer criteria. However, resource availability, transport-related issues, as well as variability in pediatric-specific training, experience, and comfort of ED clinicians working in referring hospitals may lead to delays in transfer [190]. In addition, while the ATLS course advocates that referring facilities should not obtain adjunctive diagnostic studies of injuries, which the facility does not have the capability to treat [6], a recent retrospective study showed that delayed transfer of injured children to a level I pediatric trauma center was associated with increased use of CT imaging before transfer [191]. However, the retrospective nature of the study could not clarify how many CT scans were actually obtained while waiting for a transport team or helicopter, thus being a consequence of a delay in the system, rather than a cause of delay.

Retrospective data also demonstrated that between 35% and 90% of pre- transfer head CTs in children with head trauma had to be duplicated at the referral center because of unavailable or inadequate images [192, 193]. This practice leads to increased costs, but most importantly to increased risks of cumulative radiation exposure in vulnerable children.

In order to avoid delays to definitive care of head injured children who meet local transfer criteria, and considering the increased risks and costs associated with the need of repeating CT scans at referral centers, the GDG agreed that these patients may undergo a head CT scan at the referring center only if this does not delay transfer and good quality images can be available for review by the referral neurosurgeon.

### Key action statement 15c

In centers with CT availability but without neurosurgery children with minor head trauma should be managed according to the recommendations previously provided in these guidelines for CT scan decision-making (KAS 6) and request of neurosurgical consultation (KAS 13).

ED physicians should use teleradiology, whenever available, to discuss with the referral neurosurgical unit the transfer of children with traumatic brain inury on CT.

#### Action statement profile: KAS 15c

**Table:** 

Aggregate evidence quality	B
Benefits	Optimization of decision-making on inter-hospital transfer. Cost savings and reduction of transfer-related discomfort for patients (and their families) who can be safely managed at the referring hospital
Risk, harm, cost	Cost of real-time digital imaging system platforms for health care institutions
Benefit-harm assessment	Benefits outweigh harms
Values judgments	In making this recommendation the GDG also considered evidence from adult studies
Intentional vagueness	None
Role of patient preference	None
Exclusion	Patients with moderate-severe head trauma
Strength	Strong recommendation
Difference of opinion	None

### Accompanying text

The purpose of this statement is to provide guidance on the modality of neurosurgical consultation for children with minor head trauma who are diagnosed with intracranial injury on CT at a center with CT capability but without neurosurgery.

Children with minor head trauma have a very low risk of ciTBI [3] and the great majority can safely be managed in centers with CT scan capability but without neurosurgery. For patients who undergo a CT scan that shows a traumatic intracranial injury other than an isolated linear skull fracture a neurosurgical consultation with the referral neurosurgical unit is required to guide transfer decisions. Depending on the type and extent of injury some patients will need to be immediately transferred to the referral center (for urgent neurosurgical intervention, neurointensive monitoring or high likelihood of deterioration) while others may be safely managed in the referring center. Direct visualization of CT scan images by the referral neurosurgeon allows for optimization of transfer decision based on a more accurate understanding of the patient’s lesions. The electronic transmission of digitalized medical images (teleradiology) has been used and studied since the 1990’s as a tool to improve decision making for inter-hospital transfer of head injured patients [194–196].

Several observational studies, mostly retrospective and mainly including adult patients have assessed the use of teleradiology for the management of inter-hospital transfer of patients with head trauma [194–209]. Despite the differences in methodological quality, all studies consistently found a beneficial effect of teleradiology in improving decision-making on transfer. The use of teleradiology was associated with a reduced number of unnecessary transfers, with savings in costs and resource utilization, and a reduced number of adverse events during transfer.

Although smartphone and personal digital assistant devices could be used for the electronic transmission of CT images [208, 209], the most common image transmission modality are nowadays computerized image transfer systems, which link the referral center to several referring hospitals. This technology is increasingly available for inter-hospital communication in Italy and the GDG encourages its widespread diffusion in the whole country.

### Key action statement 15d

ED physicians working in centers with CT capability but without neurosurgery should transfer to referral pediatric centers children with minor head trauma who need clinical observation whenever resources for appropriate clinical observation are not available in the referring center.

#### Action statement profile: KAS 15d

**Table:** 

Aggregate evidence quality	X
Benefits	Appropriate utilization of resources for patient safety. Likely avoidance of unnecessary radiation and costs of CT scan.
Risk, harm, cost	Costs of transfer
Benefit-harm assessment	Benefits outweigh harms
Values judgments	None
Intentional vagueness	None
Role of patient preference	None
Exclusion	None
Strength	Strong recommendation
Difference of opinion	None

### Accompanying text

The purpose of this statement is to provide guidance on the management of children with minor head trauma who need clinical observation in centers with CT availability, but without neurosurgery and lack of appropriate resources for adequate monitoring of these patients.

Children with minor head trauma may require monitoring during observation as an alternative to immediate CT scan, for persistent symptoms or personal risk factors despite a negative CT scan, or following discussion with a neurosurgeon for intracranial injuries that can be managed conservatively. Decision on the destination facility will be guided by neurosurgical advice for patients with positive CT scan amenable of conservative treatment. For patients who have not undergone a CT scan physicians should follow local transfer guidelines to determine the most appropriate referral center.

### Key action statement 15e

ED physicians working in centers with CT capability but without neurosurgery should transfer to referral pediatric centers, preferably with pediatric neurosurgical capability, children with minor head trauma needing sedation to undergo CT scan, if no skilled staff in pediatric sedation are available at the referring center.

#### Action statement profile: KAS 15e

**Table:** 

Aggregate evidence quality	X / D with respect to characteristics of referral pediatric centers (i.e. “preferably with neurosurgical availability”)
Benefits	Appropriate utilization of resources for patient safety
Risk, harm, cost	Costs of transfer
Benefit-harm assessment	Benefits outweigh harms
Values judgments	None
Intentional vagueness	None
Role of patient preference	None
Exclusion	None
Strength	Strong recommendation
Difference of opinion	None

### Accompanying text

The purpose of this statement is to provide guidance to ED physicians working in centers without skilled staff in pediatric sedation on the management of children with minor head trauma who need sedation to undergo a head CT. Sedation may be required in uncooperative children in order to prevent movement and ensure optimal imaging quality. Children may be uncooperative because of their young age, because of fear and anxiety, for agitation or irritability secondary to their head injury or because of an underlying medical condition (e.g. pervasive or specific developmental disorders). In the era of high-speed helical CT usage there seems to be a decreasing requirement for pharmacological sedation [210–212]. Recent studies have shown that between 3% and 7% of patients who undergo head CT scan following a minor head trauma are uncooperative and require sedation [69, 70]. Sedation of pediatric patients must be performed by trained personnel with specific skills in pediatric monitoring and resuscitation for the potential for life-threatening adverse events [213]. Centers that do not have the appropriate resources to safely provide sedation to pediatric patients have to transfer these children to a facility where such resources are available.

It remains unclear whether children who need sedation for head CT scan are at higher risk of ciTBI and need transfer to a center with neurosurgical capability. The only two studies that described the use of sedation in children with minor head trauma found different rates of intracranial injury on CT in this population. The first study, which was a planned subanalysis of the multicenter PECARN head injury rule study [3, 69], found that an intracranial injury (excluding isolated linear skull fractures) was present in 8% of the 527 patients who required sedation for head CT. The number of patients who needed neurosurgery is not reported. The retrospective study by Goldwasser et al. [70] was conducted at a tertiary care Australian pediatric center and included 442 patients. Of the 28 children who required sedation, 24 had a minor head trauma (GCS of 15 in 23 patients and 14 in one patient). An abnormal CT (excluding isolated linear skull fractures) was found in 32% of the 28 patients and none required neurosurgery. The GDG felt that the small number of these children and the relatively high risk of intracranial injuries justifies the transfer of these patients to centers with neurosurgical capability, whenever this can be safely and easily achieved and following local transfer guidelines.

### Key action statement 16a

ED physicians should ensure the following criteria are met before previously-healthy children with head trauma are discharged from the ED, either after initial assessment or following a period of observation:

i. GCS 15
j. Asymptomatic or significant improvement in symptoms
k. Normal neurological exam
l. No suspicion of child abuse
m. Reliable caregivers and ability to easily return to the ED
n. No other injuries requiring admission

For children who have undergone a head CT scan

o. Normal findings or presence of isolated linear skull fracture
p. Minor intracranial injuries on CT, based on neurosurgical consultation

#### Action statement profile, KAS 16a

**Table:** 

Aggregate evidence quality	X/A
Benefits	Minimizing head injury related complications (delayed diagnosis of intracranial injury) by ensuring safe discharge
Risk, harm, cost	None
Benefit-harm assessment	Benefits outweigh harms
Values judgments	Provision to families of detailed discharge instructions on warning signs that warrant further assessment is essential for safe discharge. The evidence supporting single items of the discharge criteria list is reported in the text.
Intentional vagueness	None
Role of patient preference	Parents preference should be considered
Exclusion	Children with bleeding disorders, comorbid conditions
Strength	Strong recommendation
Difference of opinion	None

### Accompanying text

The purpose of this statement is to offer guidance on safe discharge criteria for children presenting with head trauma to the ED.

Some of the recommended discharge criteria reflect general principles intrinsic to good clinical practice and can be classified, according to the GDG, as “exceptional situations where validating studies cannot be performed and there is clear preponderance of benefit over harm” (level of evidence X). Other criteria are supported by available scientific evidence. The following criteria are believed to belong to the former group: “no suspicion of child abuse”, “reliable caregivers and ability to easily return to the ED”, “no other injuries requiring admission” and decision to discharge from the ED children with “minor intracranial injury on CT, who meet the other discharge criteria, based on neurosurgical advice”. The other discharge criteria are supported by the key body of evidence highlighted below.

The PECARN head injury rule study identified previously-healthy children at very low risk of clinically important TBI who can be safely discharged without head CT based on normal mental status, absence of signs of skull fractures and absence of specific symptoms [3]. In two recent prospective studies assessing the effectiveness of clinical observation as an alternative to initial CT scan, children who were discharged following improvement of symptoms or their resolution during observation were found to have an extremely low risk of delayed diagnosis of ciTBI [111, 112]. A large retrospective population based study showed that children with a GCS of 15, normal neurologic examination and no prolonged loss of consciousness or amnesia following their head trauma have a risk between 0.01%– 0.07% of delayed diagnosis of intracranial injury [169]. Two recent planned secondary analyses of the PECARN head injury parent study demonstrated that previously healthy children with either a completely normal CT or with an isolated linear skull fracture on CT after minor head trauma have a very low risk of evolving other traumatic findings noted in subsequent imaging studies or requiring neurosurgical intervention [172, 214].

Similar sets of ED discharge criteria have been recommended by recent high-quality guidelines on the management of children with head trauma [94, 175, 215].

While the target of this recommendation are previously-healthy children, physicians should use clinical judgment when deciding to discharge from the ED children with comorbid conditions.

Provision to patients and their caregivers of detailed discharge instructions on warning signs of possible intracranial complications that should warrant prompt reassessment in the ED is a key component of a safe discharge strategy.

### Key action statement 16b

ED physicians should give verbal and printed discharge advice to children with head trauma and their caregivers upon discharge from the ED or ED observation unit.

The advice given should include:

e. Signs and symptoms that warrant medical review
f. The recommendation that a responsible adult should monitor the patient for the first 24 h after trauma
g. Details about the possibility of persistent or delayed symptoms following head trauma and whom to contact if they experience ongoing symptoms
h. Information about return to school and return to sports for children who sustain a concussion

#### Action statement profile, KAS 16b

**Table:** 

Aggregate evidence quality	B
Benefits	Limiting time to diagnosis of delayed complication; limit persistent post concussive symptoms or risk of re-injury in children who sustain a concussion
Risk, harm, cost	Cost of resources to implement the strategy, cost of information leaflets
Benefit-harm assessment	Benefits outweigh harms
Values judgments	None
Intentional vagueness	None
Role of patient preference	Literature on patient views and preferences on discharge advice was considered
Exclusion	None
Strength	Strong recommendation
Difference of opinion	None

### Accompanying text

The provision of appropriate information for patients and their caregivers upon discharge from the ED following a head trauma is important to ensure prompt recognition of warning signs for a possible initially missed and/or evolving intracranial injury. In addition, the risks related to concussive injuries (i.e. risk of re-injury if untimely return to sport or persistent post-concussive symptoms) require advice to limit further morbidity resulting from inappropriate management [104, 215].

In the latest update of the NICE guidelines [94] the NICE GDG conducted a systematic review to clarify what information and support patients with head injury say they want and what discharge information should be given. They analyzed qualitative studies and surveys that reported on patients’ views and preferences on this topic and identified relevant themes. Six themes on patient information needs were identified from three qualitative studies looking at a range of ages and severities of injury [216–218]. The data of six surveys were used to support the themes and to provide general information on the use of patient discharge advice and whether patients understood or remembered this advice [216, 219–223]. The themes identified were: need for immediate information regarding the head injury; knowing when to return to the ED; need for information concerning return to everyday activities; return to sport; information about the recovery process, and age appropriate information. Based on these themes and on an accurate analysis of the retrieved evidence described in the NICE full-text guidelines, the NICE GDG recommended for both verbal and written advice to be provided to patients and their families. The advice for children should include information on the nature and severity of the injury, the risk factors that mean patients need to return to the ED, the need for a responsible adult to monitor the patient in the first 24 h after injury, the different trends in recovery and the possibility of persistent or delayed symptoms, contact details of community and hospital services in case of delayed complications, and information about return to school and return to sport.

Provision of return to learn and return to play advice is key to facilitate symptoms resolution and reduce the risk of re-injury in children who sustain a concussion. According to the 2012 Zurich Consensus Statement on Concussion in Sport concussion is a subset of head injury characterized by a functional disturbance of the brain, rather than a structural injury, following a direct or an indirect trauma to the head, the latter defined as the result of an ‘impulsive’ force transmitted to the head [9]. More than 50% of concussions in children younger than 15 years are sport related [224]. Concussion typically results in a short-lived impairment of neurological function that resolves spontaneously. However, it has been reported that approximately 25–35% of children may still experience physical, cognitive, emotional or behavioral post-concussive symptoms at one month after injury. These symptoms may significantly affect the quality of life of patients and their families by interfering with return to their normal daily activities (school, sport and social participation) [104]. Current guidelines agree that the cornerstone of concussion management is early recognition, removal from play, rest until cerebral recovery and graduated return to cognitive and physical activity [9, 215, 225, 226]. Premature return to school or high cognitive activity can lead to prolonged symptoms, and early return to play with ongoing symptoms and slowed protective reactions puts the athlete at risk of further injury and exacerbation of concussive symptoms. Although many recommendations are based on expert consensus the body of evidence on concussion is rapidly growing and is progressively being incorporated into most recent guidelines [215, 227]. While awaiting for the upcoming Berlin Consensus Statement on Concussion in Sport, to be held in October 2016, the GDG advises for a graduated, step-wise return to school and to sport, to be individualized based on the child symptoms evolution over time, monitored by a health care professional. While return to school (full cognitive recovery) should always precede return to sport, the child should rest mentally and physically for a few days after a concussion. Return to learn and to sport should start with light cognitive/physical activity that will be increased gradually as long as symptoms do not get worse [9]. The process may last a few days to several weeks based on the child’s ability to tolerate increased cognitive load and physical activity.

Various studies have shown variability in the quality and clarity of discharge instructions provided in the ED and highlighted insufficient advice on post-concussion features and recovery [226, 228–231]. Given the risks associated with untimely return to both physical and cognitive activity after concussion, improved awareness and standardization of disposition upon discharge from the ED are important for the management of children with concussion. Recent studies showed good compliance of patients with return to sport instructions provided at discharge from the ED [232, 233].

## Conclusions

This document provides updated evidence-based guidance on i) the initial assessment and stabilization; ii) the diagnosis of clinically important traumatic brain injury; and iii) the management and disposition of children presenting to the ED with head trauma, within the first 24 h of their injury. The assessment and management of cervical spine injuries that may be associated with head trauma and the management of children with a history of bleeding disorder are not covered by these guidelines.

## Abbreviation

ATLS: Advanced trauma life support
AVPU scale: Alert, vocal, pain, unresponsive scale
CI: Confidence interval
ciTBI: Clinically important traumatic brain injury
CPR: Clinical prediction rules
CT: Computed tomography
ED: Emergency department
EPALS: European pediatric advanced life support
GCS: Glasgow coma scale
ICP: Intracranial pressure
ICU: Intensive care unit
NIRS: Near infrared spectroscopy
PALS: Pediatric advanced life support
RCT: Randomized controlled trials
TBI: Traumatic brain injury
US: Ultrasound
XR: X-rays
